# A Natural Major Module Confers the Trade‐Off between Phenotypic Mean and Plasticity of Grain Chalkiness in Rice

**DOI:** 10.1002/advs.202506242

**Published:** 2025-08-22

**Authors:** Juncheng Zhang, Yu Du, Pengkun Xu, Luyu Zhong, Zhi Li, Wenran Zhu, Yicheng Li, Bo Cheng, Xinyuan Chang, Yawei Fan, Yibo Li

**Affiliations:** ^1^ National Key Laboratory of Crop Genetic Improvement and National Centre of Plant Gene Research (Wuhan) Huazhong Agricultural University Wuhan 430070 China; ^2^ Hubei Hongshan Laboratory Wuhan 430070 China; ^3^ College of Life and Environmental Science Hangzhou Normal University Hangzhou 311121 China

**Keywords:** a natural major module, grain chalkiness, rice, trade‐off between phenotypic mean and plasticity

## Abstract

A critical challenge in crop breeding is the trade‐off between improving the mean of an important trait and maintaining its phenotypic plasticity. Grain chalkiness is a key cereal grain‐quality trait highly susceptible to environments. However, the genetic and molecular mechanisms controlling the trade‐off between phenotypic mean and plasticity of grain chalkiness remain unknown. Here, utilizing comprehensive genome‐wide association studies on ten grain chalkiness traits over five years in a mini‐core collection, substantial phenotypic plasticity of grain chanlkiness is found, which declines during modern breeding. A general trade‐off between phenotypic mean and plasticity of grain chalkiness is demonstrated, which are controlled by distinct genetic architectures revealed through a detailed QTL atlas. High temperature and wide grain significantly increase grain chalkiness mean but decrease its plasticity and genetic dissection, representing two major external drivers for the trade‐off. Two key quantitative trait genes *MPC5* and *GCP6* are identified to control this trade‐off. The transcription factor GCP6 biochemically and genetically inhibits *MPC5* expression, forming a key module that confers the mean‐plasticity trade‐off of both grain chalkiness and width. Finally, minimal marker sets for molecular breeding accounted for two thirds of grain chalkiness variation. These findings elucidate the genetic architecture of the mean‐plasticity trade‐off in grain chalkiness and offer a proof‐of‐concept breeding strategy to simultaneously optimize both phenotypic mean and plasticity in crop improvement.

## Graphical abstract

This study reveals the genetic and molecular mechanisms controlling the general trade‐off between phenotypic mean and plasticity of grain chalkiness in rice and identifies two major external drivers (high temperature and wide grain) and a natural major module (GCP6‐*MPC5*) that control this trade‐off. This work holds significant implications for a proof‐of‐concept breeding strategy in integrating both phenotypic mean and plasticity.

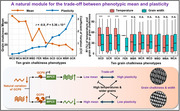

## 1 Introduction

Phenotypic plasticity enables a given genotype to produce different phenotypes under varying environments, which is fundamental for adaptation.^[^ ^1^, ^2^ ^]^ Understanding its genetic and environmental basis is particularly critical for agriculture facing climate changes.^[^ ^3^ ^]^ In phenotypic plasticity studies, phenotypic mean refers to the baseline trait value of a genotype in a reference environment. Linear plasticity quantifies the magnitude of trait change across environments (i.e., the slope of a reaction norm), while nonlinear plasticity captures deviations from linear responses (e.g., curved or threshold‐based trait shifts) reflecting disproportionate sensitivity to environmental shifts.^[^ ^4^, ^5^ ^]^ These distinctions are critical for interpreting adaptive capacity under variable conditions. Both mean and plasticity components, alongside genotype‐by‐environment interactions (G × E), determine trait performance and adaptive capacity across variable conditions.^[^ ^6^, ^7^ ^]^ Critically, a fundamental trade‐off exists between phenotypic mean and plasticity:^[^ ^2^, ^6^, ^7^ ^]^ increased phenotypic mean often comes at the cost of plasticity, and vice versa.^[^ ^2^, ^5^ ^]^ This trade‐off presents a major challenge for crop breeding, affecting both trait performance and environmental adaptation. However, the genetic and molecular mechanisms controlling this mean‐plasticity trade‐off, especially for grain quality and yield under complex field condition, remain largely unclear in crops.

Grain quality directly impacts nutrition, economic value and farmer income globally, especially in cereal‐dependent regions like Asian and African.^[^ ^1^, ^8^ ^]^ Adverse environmental factors, such as high temperatures, cause significant yield losses and severely compromise grain quality, threatening food security.^[^ ^9^, ^10^ ^]^ Grain chalkiness, a chalky texture of endosperm, is a universal critical quality trait in major cereals (known as opaque in maize and grain hardness in wheat).^[^ ^11^, ^12^, ^13^ ^]^ In rice, it seriously deteriorates grain appearance quality, milling quality, head rice yield and eating and cooking quality, market value, and farmer income.^[^ ^14^, ^15^ ^]^ Rice grain chalkiness is a complex polygenic trait highly susceptible to environmental factors,^[^ ^15^, ^16^, ^17^ ^]^ displaying strong phenotypic plasticity. This plasticity complicates reliable phenotyping and hinders the application of quantitative trait loci (QTL) in breeding. Given the increasing frequency of high temperature globally^[^ ^18^ ^]^ and its potential effect in increasing grain chalkiness,^[^ ^15^, ^17^ ^]^ reducing grain chalkiness is a critical global challenge for rice production. Although numerous QTL have been identified by linkage and GWAS mapping over the past two decades,^[^ ^19^, ^20^, ^21^ ^]^ only three QTGs (quantitative trait genes) with partial effects have been cloned.^[^ ^11^, ^22^, ^23^, ^24^ ^]^ The global genetic architecture and molecular mechanisms underlying the phenotypic mean and plasticity of grain chalkiness remain unknown. Grain size/shape is a critical determinant of appearance quality and stable yield in rice. Among the key genetic regulators of grain size in rice, the gene *GW5* (also known as *GSE5*), independently cloned by multiple research groups, plays a master role in negatively controlling grain width.^[^ ^25^, ^26^ ^]^ The loss‐of‐function allele (*gw5*) increases grain width and yield but elevates grain chalkiness (≥30%) due to disrupted starch packing in endosperms.^[^ ^26^ ^]^ Grain size is thought to confer pleiotropic effects on grain‐quality trait, but the molecular mechanism is unclear.^[^ ^27^, ^28^ ^]^

Breeding superior rice cultivars with low chalkiness and high environmental adaptability urgently requires identifying major QTGs governing the trade‐off between grain chalkiness mean and plasticity and elucidating their mechanisms. Here, we revealed this fundamental trade‐off controlled by distinct genetic architectures through compiling a detailed QTL atlas based on ten grain chalkiness phenotypes assessed over five years in 533 diverse rice accessions. We identified a master QTG, *MPC5*, which inversely regulates mean and plasticity with large effect on the trade‐off. We further elucidated *GCP6*, a critical regulator of grain chalkiness plasticity under high temperature and grain size. GCP6 functions by binding the *MPC5* promoter and inhibiting its expression, forming a natural major module conferring the phenotypic trade‐off. Finally, we established minimal marker sets and an optimal prediction model for molecular breeding by incorporating the plasticity information. Our findings provide profound insights into the mechanisms underpinning the trade‐off of an important agronomic trait and guidance for breeding superior quality rice through integrating them.

## 2 Results

### 2.1 Extensive Natural Variation and Genetic Trade‐Off between Phenotypic Mean and Plasticity of Grain Chalkiness

**Figure 1:**
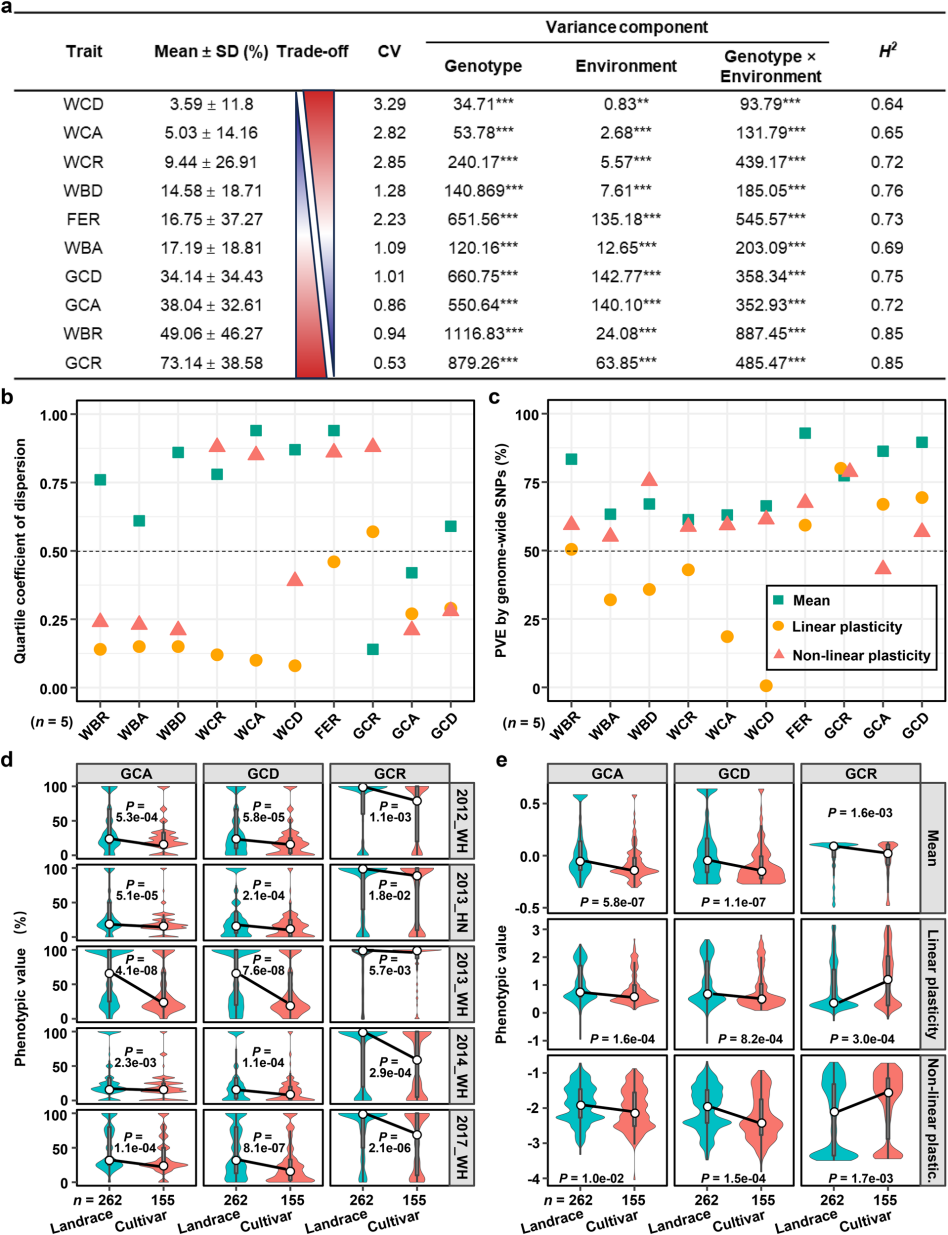
The trade‐off features of phenotypic mean and plasticity of rice grain chalkiness over five‐year trials and during rice breeding improvement. a) The mean phenotypes and variance components of the 10 grain chalkiness traits in five‐year environments. We classified three types of grain chalkiness (white belly, white core and floury endosperm) based on their location within endosperms, and measured three grain chalkiness indicators (rate, area, and degree) for each type. SD, standard deviation; CV, coefficient of variation. ***, 0 < *P* < 0.001; **, 0.001 < *P* < 0.01. b,c) Quartile coefficients of dispersion and PVE estimates of the phenotypic mean, the linear and nonlinear plasticity of the 10 grain chalkiness phenotypes in rice. The number of environments used to calculate plasticity is given in parentheses (*n* = 5). d,e) Trends of GCA, GCD and GCR (d) and phenotypic means, the linear plasticity and nonlinear plasticity (e) during rice breeding improvement identified using the phenotypes under five environments. The sample number of landrace and cultivar accessions is 262 and 155, respectively. Statistics was done using two‐tail student's *t*‐test. WBR, grain white belly rate; WBA, grain white belly area; WBD, grain white belly degree; WCR, grain white core rate; WCA, grain white core area; WCD, grain white core degree; FER, floury endosperm rate; GCR, grain chalkiness rate; GCA, grain chalkiness area; GCD, grain chalkiness degree.

Using a globally diverse mini‐core collection of 533 rice accessions,^[^ ^29^, ^30^ ^]^ we assessed 10 grain chalkiness phenotypes over five years in multiple environments (**Figure** **1**a; Table S1, Supporting Information): white core degree (WCD), white core area (WCA), white core rate (WCR), white belly degree (WBD), floury endosperm rate (FER), white belly area (WBA), grain chalkiness degree (GCD), grain chalkiness area (GCA), white belly rate (WBR), and grain chalkiness rate (GCR). All phenotypes exhibited substantial natural variation (0–100%) across accessions (Figure S1a–c, Supporting Information). Accession clustering highlighted environment‐driven plasticity, and correlation analyses revealed moderate‐to‐high trait correlations across environments (Figure S1d–f, Supporting Information). For example, correlation coefficient for WBR, WCR, and FER ranged from 0.32 to 0.77, 0.12 to 0.58, and 0.38 to 0.89, respectively (Figure S1d, Supporting Information). This variation provided a powerful basis to dissect the genetic basis of plasticity.

Plasticity analysis through quantifying coefficient of variation (CV) and variance partitioning showed a robust trade‐off between grain chalkiness means (3.59–73.14%) and plasticity (CV: 3.29–0.53%; *r* = −0.8, *P* = 5.36 x 10^−3^; Figure 1a). A linear mixed model confirmed significant variance from genotype, environment, and genotype‐by‐environment (G × E) interaction (*P* < 0.001; Figure 1a). Traits with high CV (>1) and predominant G × E variance exhibited higher environmental sensitivity. Broad‐sense heritability (*H^2^*) ranged from 0.64 to 0.85 (mean = 0.74; Figure 1a), confirming substantial genetic control despite environmental influence. Thus, grain chalkiness plasticity is significant, genetically controlled, and fundamentally trades off with phenotypic mean.

Bayesian Finlay–Wilkinson regression decomposed plasticity into phenotypic mean (Mean), linear (LP), and nonlinear (NLP) components, revealing divergent genetic architectures (Table S1, Supporting Information). Quartile coefficients dispersion (QCD) analysis showed higher dispersion for Mean than for LP (QCD_Mean_ > QCD_LP_ in 9/10 traits; Figure 1b), indicating adaptive divergence across environments. Genome‐wide analyses revealed high phenotypic variance explained (PVE) for Mean (61–93%), LP (60–80%), and NLP (43–79%) components (Figure 1c), consistent with high heritability (*H^2^* = 0.64–0.85; Figure 1a). Notably, the dominance of Mean‐associated variance (all PVE_Mean_ > 50%) across all traits (Figure 1c) suggests divergent selection pressures favoring stable expression over plasticity, further supporting the general mean‐plasticity trade‐off.

Comparing landraces and cultivars revealed signatures of artificial selection. Cultivars had significant lower phenotypic values for GCA, GCD, and GCR across environments (*P* < 0.01; Figure 1d), confirming reduced grain chalkiness as a primary breeding target. Cultivars generally also exhibited lower phenotypic means and plasticities than landraces, except for higher GCR plasticity component (Figure 1e). Analyses from 1950s to 1990s showed that concurrent declines in Mean, LP, and NLP of GCA and GCD, while GCR showed decreased Mean but increased LP and NLP, matching cultivar‐landrace comparisons (Figure S2, Supporting Information). This confirmed a trade‐off between GCR Mean and plasticity during breeding. In summary, artificial selection has consistently reduced the phenotypic mean and plasticity of GCA and GCD, highlighting the potential to further improve grain quality by simultaneously reducing both the Mean and plasticity of GCR.

### 2.2 Distinct Genetic Architecture of Phenotypic Mean and Plasticity of Grain Chalkiness and Minimal Marker Sets for Molecular Breeding Revealed by QTL Atlas

**Figure 2:**
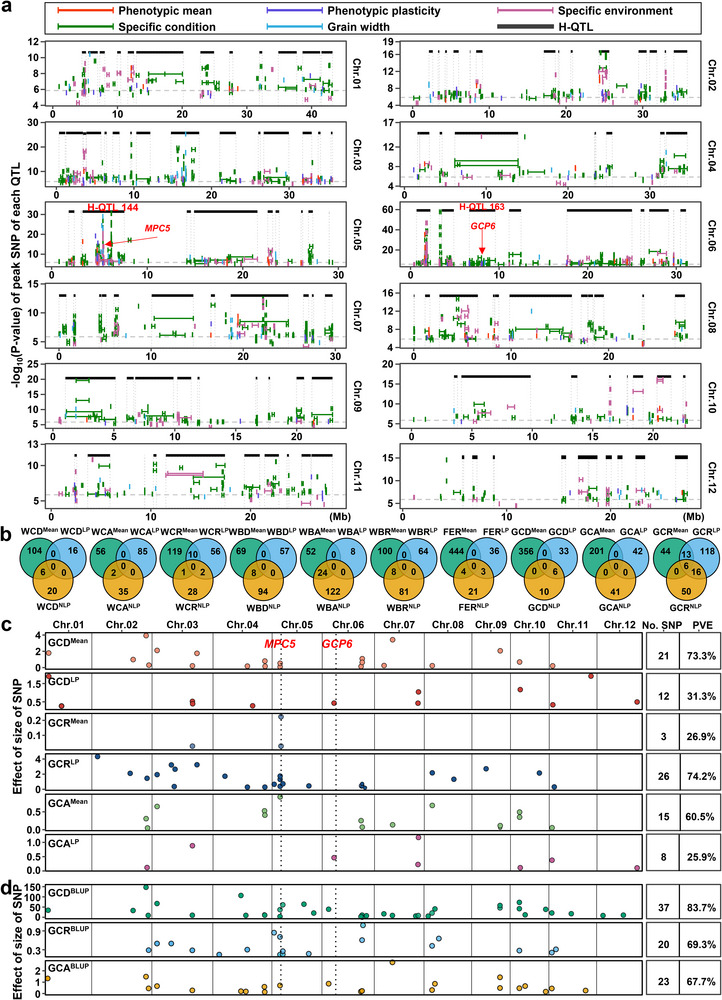
Genetic architecture underlying the phenotypic mean‐plasticity trade‐off and their diagnostic molecular markers. a) QTL atlas of phenotypic mean (77 QTL), phenotypic plasticity (107 QTL), environment‐specific phenotype (333 QTL) and condition‐specific phenotype (1070 QTL) of grain chalkiness in rice. Black rectangles showed the 129 H‐QTL across the 12 chromosomes. Grey and dashed lines indicated the threshold of GWAS. b) Comparison of the candidate genes underlying the QTL for the mean phenotype (purple circles), linear plasticity (yellow circles), and non‐linear plasticity (green circles) in the association panel. c) The minimal representative SNPs identified by LASSO regression to explain the maximum phenotypic mean and plasticity of GCD, GCR, and GCA. d) The minimal marker set determined by BLUP accounts for the most phenotypic variation of GCD, GCR, and GCA based on the combination of mean and environmental factors. Numbers and the PVEs of total SNPs were listed on the right. Each dot in the plot represents an SNP. GCR, grain chalkiness rate; GCA, grain chalkiness area; GCD, grain chalkiness degree.

To determine the comprehensive genetic architecture of rice grain chalkiness, we performed multicondition GWAS using 4131700 SNPs in 533 accessions and five‐year field phenotypes (Tables S1 and S2, Supporting Information). The identified QTL were classified into five categories: (1) phenotypic mean QTL; (2) linear and nonlinear plasticity QTL; (3) environment‐specific QTL, representing the phenotype in a given year; (4) condition‐specific QTL, referring to the phenotype of accessions grouped by genotypes of *indica*/*japonica* subspecies, *Wx* and grain‐width genes like *GW5*;^[^ ^26^, ^31^ ^]^ (5) grain width QTL. We totally identified 1702 redundant QTL, including 1587 QTL for grain chalkiness: 77 QTL for phenotypic mean, 107 QTL for phenotypic plasticity, 333 QTL for environment‐specific phenotype, and 1070 QTL for condition‐specific phenotype and 115 QTL for grain width (**Figure** **2**a; Table S2, Supporting Information). These QTL were comprehensively and differentially distributed along all chromosomes, especially for phenotypic mean and plasticity, indicating the complex genetic basis of grain chalkiness across different environments and conditions (Figure 2a; Table S2, Supporting Information). The phenotypic mean QTL are most densely distributed on chromosomes 6, 5, and 3, with counts of 16, 13, and 11, respectively, while plasticity QTL are most prevalent on chromosomes 3, 2, 1, and 6, with counts of 20, 16, 11, and 11, respectively (Figure S3a, Supporting Information). The asymmetric chromosomal distribution highlighted the genetic trade‐off of phenotypic mean and plasticity.

To resolve redundancy, we condensed 1702 QTL to 317 non‐redundant hotspot QTL (H‐QTL) (≥2 overlapping QTL; Tables S2 and S3, Supporting Information). Among these, 36 of 40 phenotypic mean H‐QTL and 54 of 68 phenotypic plasticity H‐QTL covered more than three overlapping QTL (Figure 2a; Table S3, Supporting Information). In total, 129 H‐QTL covered >3 QTL, serving as key genetic determinants of grain chalkiness in rice (Figure 2a; Table S3, Supporting Information). We identified six H‐QTL common to all five QTL categories (Figure S3b,c, Supporting Information). H‐QTL144 covered the most QTL (*n* = 147), mainly for phenotypic mean and plasticity (Table S3, Supporting Information). Excluding phenotypic mean QTL, 12 additional H‐QTL spanned four QTL categories (Figure S3d, Supporting Information). Among these, H‐QTL163 ranked second (*n* = 48 QTL), primarily regulating plasticity (Figure S3e, Supporting Information). H‐QTL144 critically controlled both phenotypic mean and plasticity, whereas H‐QTL163 predominantly regulated phenotypic plasticity. These highlights the significance of identifying causal genes within these major hotspots. Collectively, our saturated QTL and H‐QTL mapping revealed distinct genetic architectures governing phenotypic mean and plasticity of grain chalkiness under varying environments.

To further explore the genetic basis of phenotypic plasticity, we compared all the candidate genes underlying the QTL for the three sub‐phenotypes: phenotypic mean, LP, and NLP for the 10 chalkiness traits (Figure 2b). Candidate genes were defined as those located within 20‐Kb windows centered on each significant SNP.^[^ ^32^ ^]^ We found no overlapping gene among all the QTL for the three sub‐phenotypes across all the nine traits (except for GCR with only six overlapping genes among them), revealing the distinct genetic basis between phenotypic mean and plasticity of grain chalkiness in rice (Figure 2b). The non‐overlapping genomic loci governing phenotypic mean and plasticity suggest the compensatory but independent genetic architecture.

To optimize grain‐quality breeding through genome selection, we identified high‐predictivity SNPs for grain chalkiness mean and plasticity. Using LASSO regression with SNPs in 974 chalkiness‐associated genes, we filtered SNPs and removed collinear variables to build linear prediction models. These SNPs explained 26.0–74.2% PVE (mean: 48.8%) across traits (Figure 2c). Furthermore, the minimal SNP datasets revealed genetic interactions (epistasis) specific to phenotypic mean and plasticity of all traits (Figure S4 and Table S4, Supporting Information), establishing optimized minimal marker sets and prediction models for molecular breeding in rice.

Given distinct genetic architecture, combining phenotypic mean and linear plasticity SNPs significantly outperformed single‐factor PVEs (Figure S5, Supporting Information). We further applied best linear unbiased prediction (BLUP) to multienvironmental phenotypes and used LASSO to identify the minimal marker sets (Figure 2d). As a result, we identified 37, 20, and 23 representative SNPs associated with GCD, GCR, and GCA, respectively, which collectively explained 83.7%, 69.3%, and 67.7% of the phenotypic variance (Figure 2d). These results demonstrated the feasibility of using SNPs with PVEs more than 2/3 for predicting phenotypic traits, providing strong evidence for the rationality of simultaneously considering both phenotypic mean and plasticity in designing superior quality breeding strategies.

### 2.3 A Master Hotspot QTL Gene, MPC5, Drives the Mean‐Plasticity Trade‐Off of Grain Chalkiness

**Figure 3:**
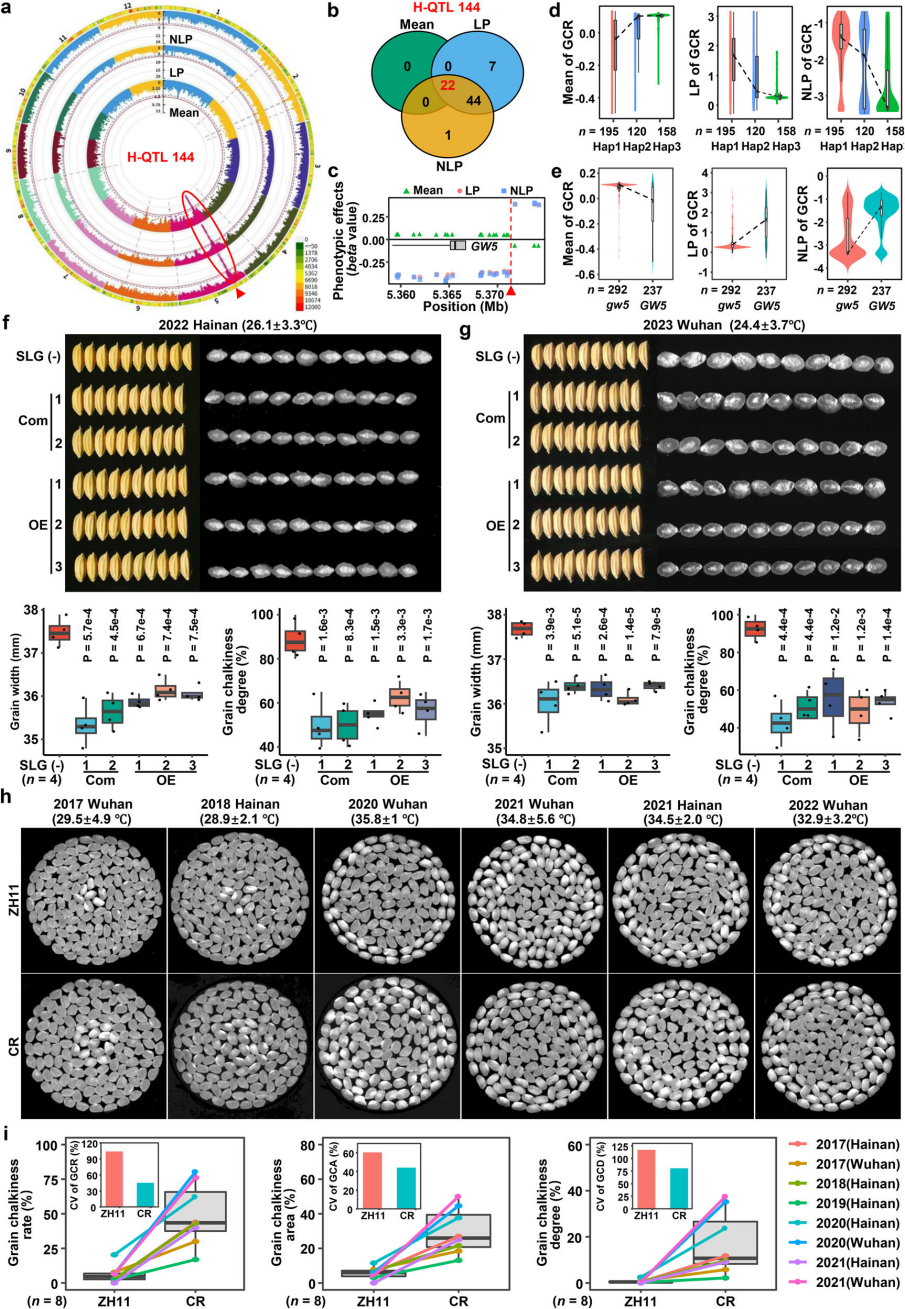
A common and major QTL gene *MPC5* confers the mean‐plasticity trade‐off of grain chalkiness. a) The important hotspot QTL (H‐QTL144) covering the most QTL with 147 QTL marked in red ellipse was identified by GWAS for both phenotypic mean and plasticity of grain chalkiness in rice. b) 22 common and most significant SNPs in the H‐QTL144 showed by Venn diagram. The 22 SNPs were filtered with *P* values < 1e‐10. c) Phenotypic effects (beta value) of the 22 SNPs estimated by the LMM model of GWAS. The red arrow indicated the breakpoint. d) Phenotypic mean and plasticity of GCR for three haplotypes composed of 22 SNPs in the mini‐core collection. e) The trends of mean, LP and NLP of GCR between *gw5* and *GW5* genotypes in the mini‐core collection. f,g) Grain chalkiness and width of two complementation and three OE lines in the SLG background in 2022 Hainan (f) and 2023 Wuhan (g). Statistics was done using two‐tail student's *t*‐test. Each transgenic line counts four samples. h) Phenotypic performances of wild type (ZH11) and knock‐out lines (CR) of *MPC5*/*GSE5*/*GW5* in six‐year environments. i) The means and CVs of grain chalkiness of wild type and knock‐out lines of *MPC5*/*GSE5*/*GW5* in eight‐year environments. The CVs were drawn inside the plots.

To identify causal genes for the major hotspot H‐QTL144 (Figure 2; Figure S3b,c, Supporting Information), we analyzed overlapping QTL and identified three representative loci, L1587 (phenotypic mean), L1580 (linear plasticity) and L1596 (nonlinear plasticity) (**Figure** **3**a; Table S3, Supporting Information). Twenty‐two shared SNPs (*P* < 1 × 10^−1^; Figure 3b) exhibited trade‐off effects (GWAS *beta*‐value contrasts) between phenotypic mean and plasticity traits (Figure 3c). SNPs left of the 5371020–5 371 694 bp breakpoint (MSU ver.6) displayed antagonistic effects (Figure 3c), consistent with haplotype analyses that Hap1–Hap3 defined by the 22 SNPs displayed opposite trend for phenotypic mean versus plasticity (Figure 3d). To validate this, we analyzed environment‐specific GCR phenotypes (phenotypic mean) (Figure S6a, Supporting Information) and the variances (phenotypic plasticity) of GCR across five environments (Figure S6b, Supporting Information) using these haplotypes, further supporting the antagonistic or trade‐off effect between GCR and its variance. These findings confirmed that H‐QTL144 is a master QTL controlling the trade‐off between the mean and plasticity of grain chalkiness rate.

The haplotype region contained a single annotated gene, *GSE5*/*GW5* (LOC_Os05g09520; Figure 3c). We next examined the trade‐off effect of *GSE5*/*GW5* using the min‐core collection and transgenic materials (Figure 3e–i). Analyses of the Mean, LP and NLP of GCR between *gw5* and *GW5* genotypes in the mini‐core collection showed that the Mean followed an opposite trend compared to both LP and NLP (Figure 3e), mirroring the H‐QTL144 pattern (Figure 3d).

To confirm the role of *GSE5/GW5* in grain chalkiness plasticity, we generated GW5 complementation and overexpression (OE) lines in the SLG background (a floury‐endosperm variety with non‐functional *gw5* allele). Both complementation and OE lines exhibited significantly reduced grain chalkiness degree and grain width across two environments of 2022 Hainan (Figure 3f) and 2023 Wuhan (Figure 3g), confirming the novel role of *GW5* in grain chalkiness. CRISPR‐knockout lines of *GW5* in ZH11 exhibited higher GCR but lower plasticity (CV) than wild‐type across eight‐year field trails (Figure 3h,i). Thus, *GSE5*/*GW5* is the causal gene underlying H‐QTL144, driving the phenotypic mean‐plasticity trade‐off. We renamed it to *MPC5* (*Mean and Plasticity of grain Chalkiness on chromosome 5*) due to its new functional role.

### 2.4 High Temperatures and Wider Grains as Two Major External Factors Increase Grain Chalkiness Mean and Decrease Its Plasticity and Genetic Detection

**Figure 4:**
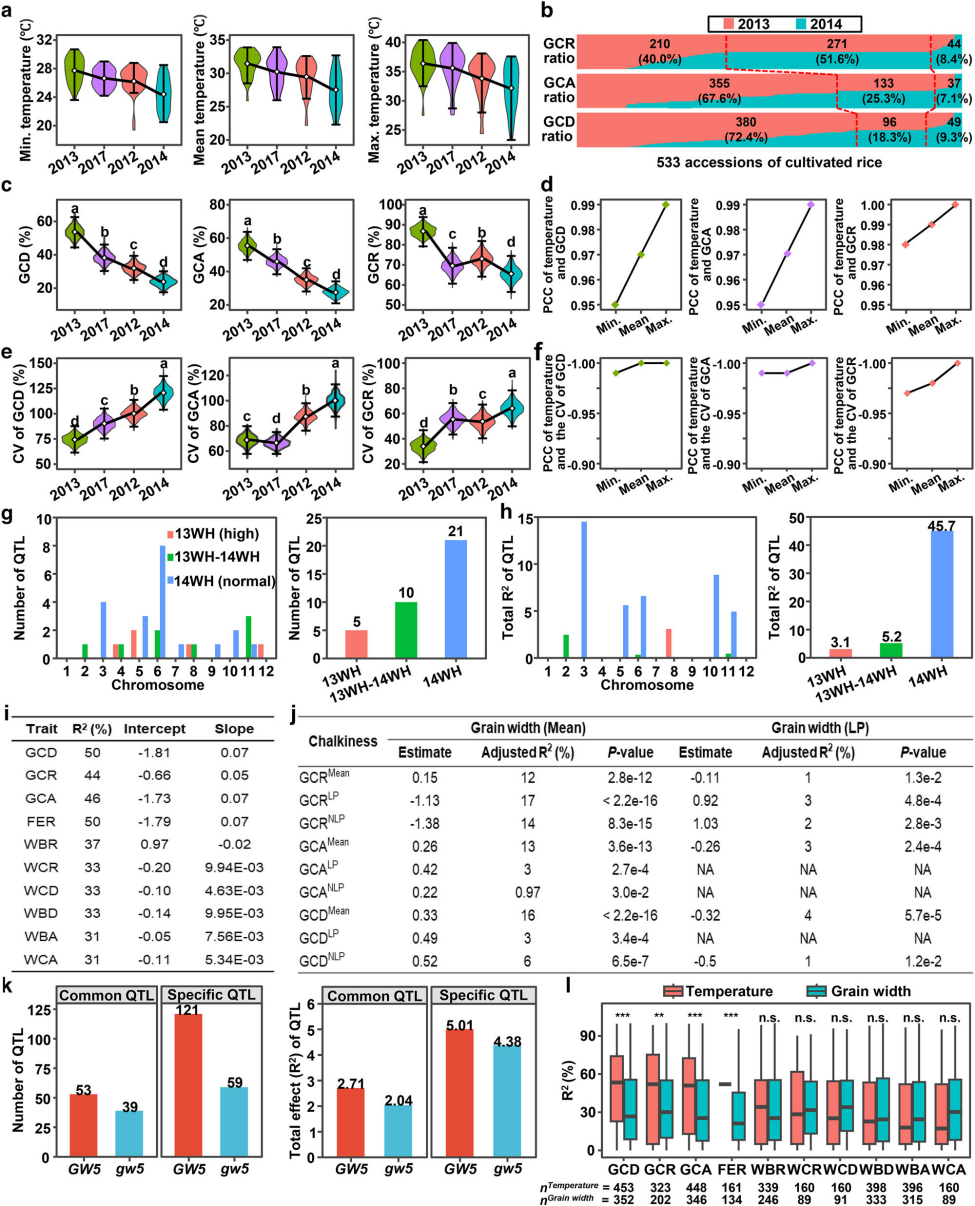
Fluctuating high temperatures and wider grains greatly increase grain chalkiness mean, depress its plasticity and genetic identification. a) Fluctuating air temperatures (the maximum, minimal, and mean values) at grain filling stages (17/07–23/08) of 2012–2014 and 2017 at Wuhan. b) The comparison of GCD, GCA, and GCR of 533 accessions between the highest temperature year of 2013 and the lowest temperature year of 2014. *y* axis is the proportion of phenotypes in 2013 (red) or 2014 (blue) to the total phenotypes in these two years. c) The three grain chalkiness phenotypes among four temperature environments. d) The Pearson correlation coefficient (PCC) of minimal, mean and maximum air temperatures with the GCD, GCA, and GCR. e) The phenotypic plasticity represented by the coefficient of variation (CV) of grain chalkiness among four temperature environments. Diamond symbol, top line and bottom line in the violins of (c, e) are the means, maximum, and minimal values, respectively. f) The PCC of minimal, mean and maximum air temperatures with the CVs of GCD, GCA, and GCR. Data used in (c, e) were simulated by sampling with replacement for 1000 times. g,h) The number (g) and genetic effects (*R* ^2^) (h) of QTL identified in 2013WH (high temperature), 2014WH (normal temperature), and 13WH‐14WH (DIFF) on each chromosome. i) Average effects of temperature at grain filling stage on grain‐chalkiness phenotypic plasticity evaluated by the PPRE model. *R* ^2^ is the PVE of temperature to grain chalkiness plasticity. Intercept and slope reflect endogenous and exogenous signals that contribute to grain chalkiness phenotypes in response to environments, respectively. j) Effects of grain‐width phenotypic plasticity on grain‐chalkiness phenotypic plasticity evaluated by a linear model. k) The number and total effect of the common and specific QTL for grain chalkiness within accessions grouped by the *GW5* and *gw5* genotypes. l) Effect evaluation of temperature and grain width on grain chalkiness by PPRE. Sample numbers used to perform PPRE analyses are given below the figure. Statistics was calculated with two‐tail student's *t*‐test. *P < 0.05, **P < 0.01, ***P < 0.001.

Rice grain chalkiness is highly sensitive to environmental factors, especially high temperature during grain filling stage. Using temperature data from four environments in Wuhan (2012–2014 and 2017; **Figure** **4**a; Table S5 and Figure S7, Supporting Information), we observed that hotter years (e.g., 2013) significantly increased chalkiness phenotypes: 40.0% (GCR), 67.6% (GCA), and 72.4% (GCD) of accessions showed elevated values versus cooler years (e.g., 2014; Figure 4b).

The trend of overall phenotypes of GCD, GCA, and GCR across four years (Figure 4c) aligned with temperature changes (Figure 4a). All traits were strongly positively correlated with air temperatures, particularly the maximum temperatures (PCC ≈ 1, *P* = 0.03) (Figure 4d). We further analyzed the coefficient of variation (CV), an indicator of phenotypic plasticity (Figure 4e), and found that CV trends were inversely correlated with both grain chalkiness means and air temperatures (Figure 4a,c,e). Remarkably, even 0.8 °C mean difference between 2017 and 2012 significantly altered mean and plasticity of GCD and GCA (Figure 4c,e), confirming the sensitivity of grain chalkiness to temperature. Air temperatures‐CV correlations were strongly negative (< −0.95; Figure 4f). Collectively, fluctuating high temperature during grain filling stage strongly promotes grain chalkiness formation while reducing phenotypic plasticity, revealing a trade‐off feature caused by a key external factor.

To quantify temperature effects on grain chalkiness plasticity, we developed DIFF score (pairwise difference using 2013 Wuhan as high‐temperature reference; Table S1 and Figure S8, Supporting Information). DIFF scores displayed substantial variation (−100% to 100%) across 533 accessions (Figure S8a–c, Supporting Information), with strong intertrait correlations (Figure S8d–f, Supporting Information). The correlation coefficients for white belly rate, white core rate and floury endosperm ranged from 0.66–0.81, 0.52–0.74, and 0.77–0.96, respectively (Figure S8d, Supporting Information), much higher than those among the 10 basic grain chalkiness traits (Figure S1d, Supporting Information), demonstrating the efficacy in capturing thermal‐driven plasticity. Thus, chalkiness variation primarily reflects external temperature differences, and DIFF serves as a sensitive indicator for dissecting genetic basis of high temperature‐driven plasticity.

We then compared the location, number and effects of 36 QTL identified for GCA, GCR, and GCD under high temperature (13WH), normal temperature (14WH), and the DIFF score (13WH–14WH) environments (Figure 4g,h). The QTL for grain chalkiness under high and normal temperatures were distributed across most chromosomes (Figure 4g), indicating the distinct genetic mechanisms. Notably, 21 QTL were identified under normal temperature, compared to only 5 QTL under high temperature (Figure 4g). The total genetic effects (*R* ^2^) of QTL identified at normal temperature (45.7%) were much higher than those at high temperature (3.1%) (Figure 4h), suggesting that high temperature hinders the genetic identification of QTL controlling grain chalkiness plasticity.

Using the phenotypic plasticity in response to the environment (PPRE) model,^[^ ^7^ ^]^ we evaluated the temperature's impact on grain chalkiness (Figure 4i). Temperature effects (*R* ^2^) during grain filling stage exceeded 31% for all traits, with an average of 38% (Figure 4i), highlighting the dominant role of environmental temperatures in grain chalkiness variation. The DIFF results further confirm that high temperature significantly impacts grain chalkiness and complicates its genetic identification in rice. Collectively, these findings confirm that high temperature is a major external factor promoting grain chalkiness formation, as well as decreasing its plasticity and genetic detection in rice.

Since the major grain‐width gene *MPC5*/*GSE5*/*GW5* pleiotropically affects both grain width and chalkiness (Figure 3), we examined the relationship between grain width and chalkiness at population level (Figure S9 and Table S1, Supporting Information). Briefly, grain width was positively correlated with the means of GCA, GCD, and GCR, but negatively correlated with the plasticity of both LP and NLP in GCR (Figure S9a, Supporting Information). This illustrated that wider grains raise baseline chalkiness but decrease environmental plasticity (LP, NLP), highlighting an agronomic trade‐off. Further analysis revealed that the phenotypic mean of grain width exhibited the strongest positive correlation with grain chalkiness mean (PCC = 0.11–0.40), but general negative correlation with plasticity in GCR (PCC = −0.38 – −0.41), consistent across the four environments (Figure S9b, Supporting Information). Notably, narrow‐grain accessions (*GW5* haplotype) followed this trend, whereas wide‐grain accessions (*gw5*) showed a different trend in GCR (Figure S9b, Supporting Information). In summary, grain width is positively correlated with the mean phenotypes of grain chalkiness but negatively correlated with the plasticity of GCR, and *MPC5* plays a crucial role in determining the trade‐off effect.

Linear modeling confirmed the mean phenotype of grain width significantly contributes to the mean of grain chalkiness, with the PVEs of 12%, 13%, and 16% for GCR, GCA and GCD, respectively, but had little effect on the LP plasticity (Figure 4j). Furthermore, QTL analyses indicated that the number and the summed PVEs of QTL were greater in *GW5* accessions compared to *gw5* accessions (Figure 4k). Collectively, these results confirm that wider grain shape promotes grain chalkiness formation and significantly affects its plasticity and genetic dissection in rice. PPRE modeling further showed temperature had a greater effect on all grain chalkiness traits than grain width (Figure 4l).

### 2.5 GCP6 Controls the Phenotypic Mean‐Plasticity Trade‐Off of Both Grain Chalkiness and Width

**Figure 5:**
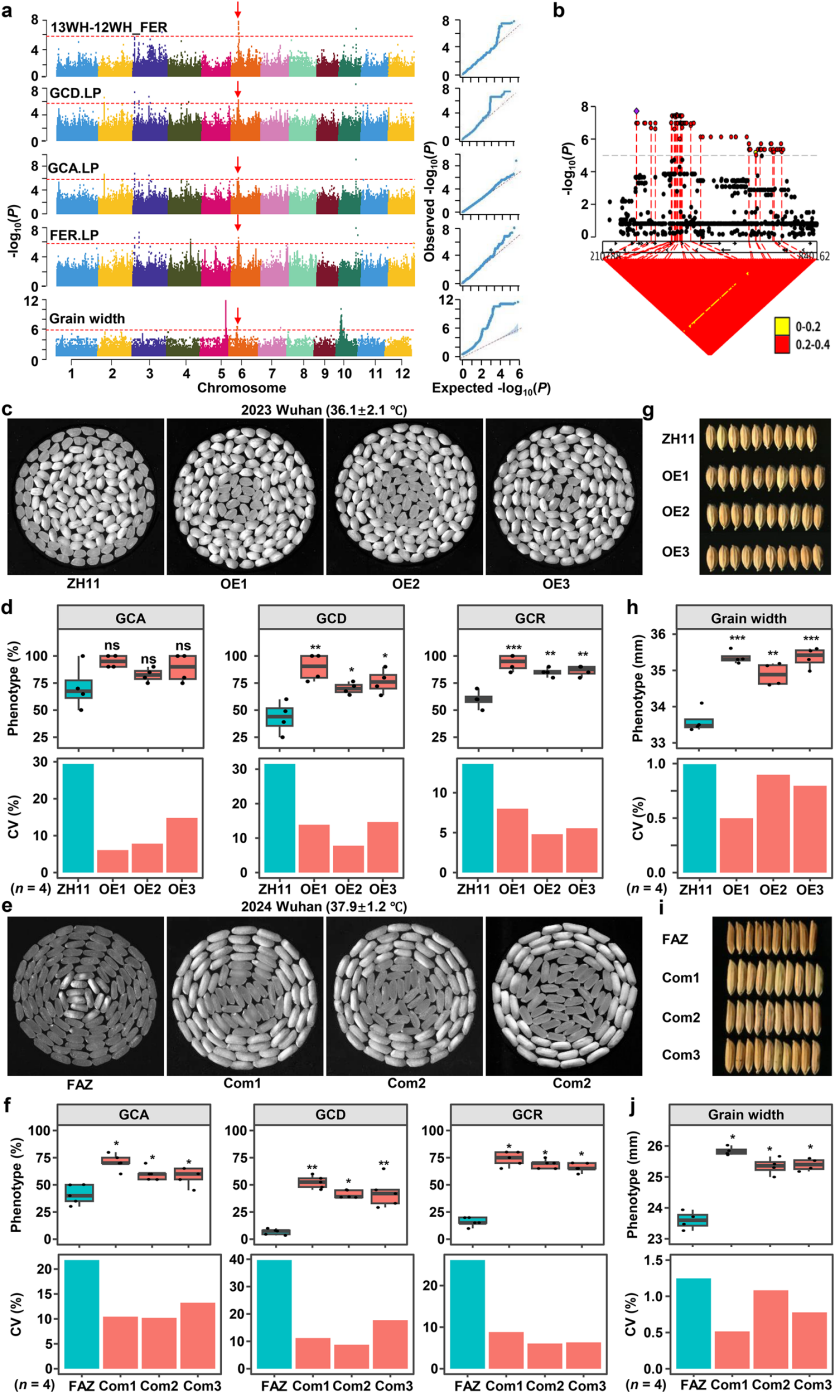
*GCP6* is the causal gene underlying the hotspot H‐QTL163. a, Colocalization of *GCP6* with QTL identified in four scenarios, including the linear plasticity of GCD, GCA, FER, grain width and the DIFF metric of 13WH‐12WH_FER. b) The LD correlation (*R* ^2^) of the SNPs in the QTL with the lead SNP. c) Grain chalkiness phenotypes of *GCP6*‐OE lines evaluated in 2023 Wuhan. d) The means and CVs of GCA, GCD, and GCR of *GCP6*‐OE lines. e) Grain chalkiness phenotypes of *GCP6* complementation lines evaluated in 2024 Wuhan. f) The means and CVs of GCA, GCD, and GCR of *GCP6* complementation lines. g) Grain width of *GCP6*‐OE lines. h) The means and the CVs of grain width of *GCP6*‐OE lines. i) Grain width of *GCP6* complementation lines. j) The means and the CVs of grain width of *GCP6* complementation lines. Significance was generated by the two‐tailed *t*‐test. *, *P* < 0.05; **, *P* < 0.01. Four replicates were used for the statistical analyses (*n* = 4).

H‐QTL163 on chromosome 6 emerged as a key locus regulating phenotypic plasticity, harboring 48 plasticity‐related QTL (Figure 2; Figure S3e, Supporting Information). This hotspot contains six plasticity QTL, 40 condition‐specific QTL, one environment‐specific QTL and one grain‐shape QTL (Table S2, Supporting Information). We focused on three linear‐plasticity QTL for GCD, GCA, and FER, and a condition‐specific QTL for FER^13WH–12WH^, which colocalized within a 691 kb region (**Figure** **5**a; Table S2, Supporting Information).

Using significant SNPs with a *P*‐value below 1e‐5, we narrowed the interval to a 98 kb region (Figure 5b). Seven of the 12 genes in the region were expressed in different rice tissues, among which only *LOC_Os06g14670*, encoding an R2R3‐MYB transcription factor, showed dominant expression in developing endosperms (Figure S10a,b, Supporting Information). Therefore, we designated *LOC_Os06g14670* as a solid candidate gene underlying H‐QTL163 and named it as *GCP6* (*Grain Chalkiness Plasticity on Chromosome 6*) hereafter. Four haplotypes of *GCP6* (H1–H4) exhibited distinct subpopulations, with H1 and H2 being the major haplotypes (Figure S10c,d, Supporting Information). H2 exhibited significantly higher grain chalkiness plasticity and higher expression in 5‐DAP endosperms (Figure S10e,f, Supporting Information).

To validate the functional role of *GCP6* in grain development, we generated CRISPR/Cas9‐mediated knockout lines (Figure S10g, Supporting Information), overexpression (OE) lines (Figure S10h,i, Supporting Information), and transgenic complementation lines in the H2 haplotype variety ZH11. *GCP6*‐OE lines exhibited significantly elevated grain chalkiness area (GCA), degree (GCD), and ratio (GCR), coupled with reduced chalkiness variability (CVs) compared to wild‐type controls (Figure 5c,d). Furthermore, genetic complementation by introducing the *GCP6* transgene from the strong H2 haplotype donor ZH11 into the weak H1 haplotype variety FAZ effectively restored these chalkiness traits, with complemented lines showing increased GCA, GCD, GCR, and decreased CVs (Figure 5e,f). These results collectively demonstrate that *GCP6* is a key regulator of grain chalkiness formation. Additionally, *GCP6*‐OE lines displayed a significant increase in grain width (Figure 5g), alongside reduced grain width variability (Figure 5h). A consistent phenotypic trend was observed in complementation lines, which showed restored grain width (Figure 5i) and stabilized width variability (Figure 5j). These data establish *GCP6* as a critical determinant of grain chalkiness and width modulation underlying the hotspot H‐QTL163 in rice.

**Figure 6:**
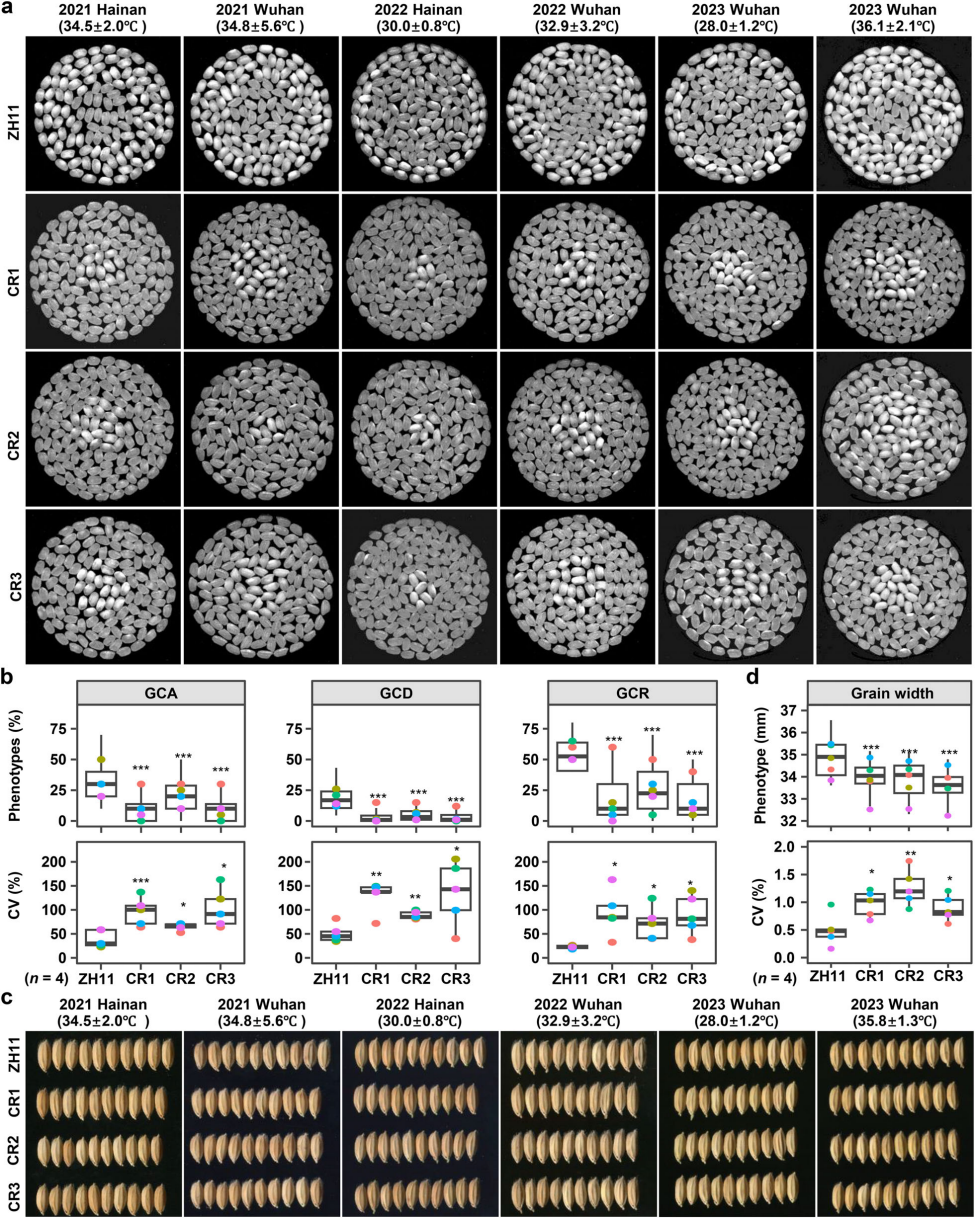
*GCP6* confers the phenotypic plasticity of both grain chalkiness and width. a) Grain chalkiness phenotypes of *GCP6* mutant lines planted under five‐year field trials with diverse natural temperature conditions. b) The means and their CVs of GCA, GCD, and GCR of the *GCP6* mutant lines. c) Grain width phenotypes of *GCP6* mutant lines under five‐year field trials with diverse natural temperature conditions. d) The means and its CVs of grain width of *GCP6* mutant lines. Four plants per mutant were assayed for grain chalkiness (b) and grain width (d) (*n* = 4). Significance was generated by the two‐tailed *t*‐test. *, *P* < 0.05; **, *P* < 0.01.

Field trials across six‐environments (three years × two thermal‐contrasting sites) consistently confirmed the phenotypic effects of *GCP6* using knockout lines (**Figure** **6**). Three independent knockout lines reduced grain chalkiness area (GCA), degree (GCD), and ratio (GCR), but increased chalkiness variability (CVs) versus wild type (Figure 6a,b). Furthermore, these mutants also decreased grain width (Figure 6c) while increasing grain width‐related CVs (Figure 6d), demonstrating pleiotropic regulation on grain morphology.

These data definitively confirmed *GCP6* as the causal gene for H‐QTL163. More importantly, the antagonistic pleiotropy between phenotypic mean (grain chalkiness and grain width) and their variabilities (CVs) mechanistically established *GCP6*’s role in modulating the trade‐off between phenotypic means and plasticity of both grain chalkiness and width in rice.

### 2.6 GCP6 Transcriptionally Represses MPC5 Expression to Regulate the Trade‐Off of Grain Chalkiness Plasticity

**Figure 7:**
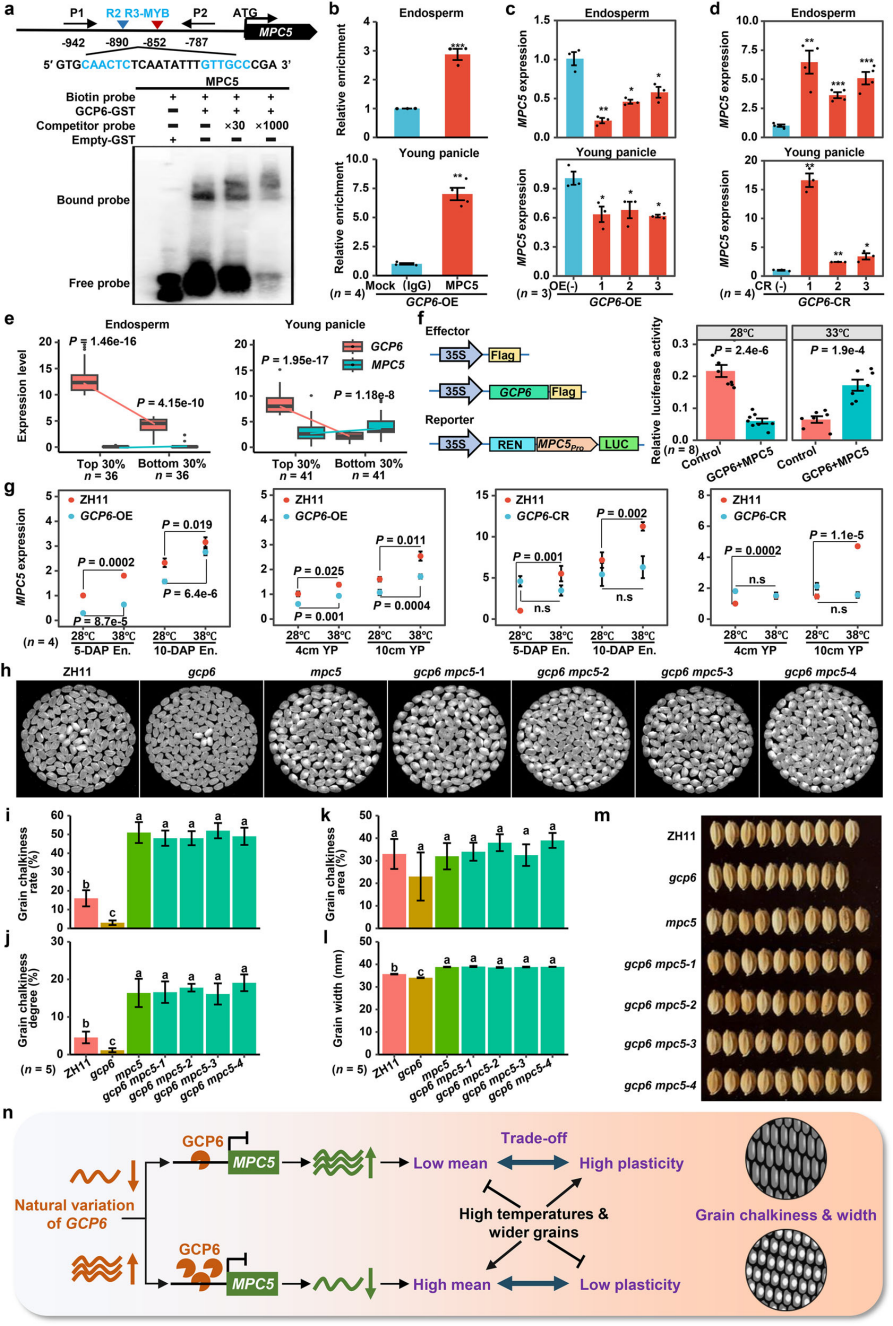
GCP6 represses *MPC5* expression to positively regulate both grain chalkiness and width. a) EMSA analysis for GCP6 binding to R2R3‐MYB in *MPC5* promoter. b) ChIP‐qPCR assays of *MPC5* in 5‐DAP endosperms (upper) and 4‐cm young panicles (lower) of *GCP6* over‐expression lines. Primer sets of P1 and P2 were indicated in (a). The data show relative enrichment of DNA precipitated with Flag antibody to those treated with IgG (set to 1). The fragments in an exon of *MPC5* and promoter of *Ubi* (*OsUBQ5*) were used as controls. Data are representative means ± SE (*n* = 4). c,d) Expression of *MPC5* in 5‐DAP endosperms (upper) and 4‐cm young panicles (lower) of *GCP6* over‐expression lines (c) and *GCP6* mutant lines (d). Three and four biological replicates were used for each OE (c, *n* = 3) and CR line (d, *n* = 4), respectively. e) Expression levels of *GCP6* and *MPC5* in 5‐DAP endosperms and 4‐cm young panicles of top 30% and bottom 30% accessions of *GCP6* expression. f) Transcriptional activity analysis of GCP6 on *MPC5* using the DLR assays under different temperatures. In the luciferase assay, the *GCP6* gene was utilized as the effector to investigate its regulatory effects, while the *MPC5* gene was employed as the reporter to measure the transcriptional activity. The relative luciferase activity (LUC/REN) was measured after a 3‐d coexpression period at 28 °C, followed by an additional 2.5 d at 28 °C and a subsequent half day at 33 °C. Data are shown as means ± SE (*n* = 8). The *P* values denote significance compared with the negative control by two‐tailed *t*‐test. g) Expression of *MPC5* in *GCP6* mutant and overexpression lines (5‐ and 10‐DAP endosperms, 4 and 10‐cm young panicles) sampled at normal (28 °C) and high temperature (38 °C). Four replicates per line per condition were used for the assay (*n* = 4). h–k) Grain chalkiness phenotypes of *gcp6* and *mpc5* single mutants and *gcp6 mpc5* double mutants. l,m) Grain width phenotypes of *gcp6* and *mpc5* single mutants and *gcp6 mpc5* double mutants. The same letter above the bars indicates lack of significant differences according to Tukey–Kramer multiple comparisons test (one‐way analysis of variance (ANOVA) between groups). Five replicates were used for the statistical analyses (*n* = 5). *P* < 0.05 was considered as significance. n) A working model of the GCP6‐*MPC5* module in regulating the mean‐plasticity trade‐off of both grain chalkiness and width in rice.

Since *GCP6* (R2R3‐MYB transcription factor) and *MPC5* exhibits similar mean‐plasticity trade‐off functions, we hypothesized a potential regulatory crosstalk. Of fourteen R2R3‐MYB binding motifs in the *MPC5* promoter, two motifs (CAACTC and GTTGCC, −880 and −859 bp) overlapped with chromatin accessibility peaks^[^ ^33^, ^34^, ^35^ ^]^ (**Figure** **7**a; Figure S11a, Supporting Information). An electrophoresis mobility shift assay (EMSA) using biotin‐labeled probes containing these motifs confirmed that GCP6 binds to this *MPC5* promoter region (Figure 7a). Chromatin immunoprecipitation‐qPCR (ChIP‐qPCR) using 5‐DAP endosperms and 4‐cm young panicles from *GCP6*‐OE lines showed significant binding of GCP6 to the *MPC5* promoter in vivo (Figure 7b). These results confirmed that *MPC5* is a direct target of GCP6.

To further validate this regulatory relationship, we measured *MPC5* expression in *GCP6* mutant and OE lines (Figure 7c,d). *MPC5* expression was significantly reduced in both 5‐DAP endosperms and 4‐cm young panicles of *GCP6* overexpression lines (Figure 7c), and was significantly upregulated when *GCP6* was nonfunctional (Figure 7d). Furthermore, the expression of *MPC5* and *GCP6* in endosperms and young panicles were negatively correlated across all accessions (Figure S11b, Supporting Information), and in accessions with extreme *GCP6* expression levels from the mini‐core collection (Figure 7e). These findings reveal a significant negative coexpression pattern for these genes in rice natural populations, consistent with the effects observed in *GCP6* mutant and OE lines. Thus, *GCP6* acts as a transcriptional suppressor of *MPC5*.

To explore the regulatory relationship under different temperature conditions, we performed dual luciferase reporter (DLR) assays. Co‐transformation of *GCP6* with *MPC5* promoter resulted in lower promoter activity at 28 °C but higher activity at 33 °C (Figure 7f), indicating that the transcriptional inhibition of *MPC5* by GCP6 is weakened under high temperature. Similarly, the expression of *GCP6* and *MPC5* was down‐ and up‐regulated from 28 °C to 36 °C, respectively, in both endosperms and young panicles of the mini‐core collection (Figure S11c,d, Supporting Information) and in endosperms of two varieties, ZH11 and MH63 (Figure S11e,f, Supporting Information). Furthermore, *MPC5* was upregulated by high temperature in ZH11 and *GCP6*‐OE lines, but remained unchanged in *GCP6* mutants (Figure 7g), indicating that GCP6 is required for *MPC5* expression in response to temperatures. Collectively, these results confirmed the negative regulatory role of GCP6 on *MPC5* in differential response to high temperatures.

Linear regression further revealed opposite effects of *GCP6* and *MPC5* on both grain‐chalkiness mean and plasticity, with *GCP6* having a positive effect and *MPC5* having a negative one (Figure S12a, Supporting Information). Their combined PVEs exceeded single‐gene effects (Figure S12b, Supporting Information). Furthermore, the four haplotypes of *GCP6* and *MPC5* in the mini‐core collection showed significant differences in all nine grain chalkiness plasticity traits and one grain width trait, and the *gcp6*‐*MPC5* genotype minimized chalkiness (Figure S12c, Supporting Information). This is consistent with breeding selection signatures that landrace‐cultivar comparisons of population differentiation index (Fst) indicated modern rice breeding imposed strong selection at *GCP6* (Fst > 0.15) and *MPC5* (Fst > 0.125) (Figure S12d, Supporting Information). These findings align with the observed reduction in phenotypic means and plasticities of grain chalkiness traits in cultivars compared to landraces (Figure 1d,e), suggesting these genes may underlie artificial selection documented in breeding programs. The progressive diversity loss of the two genes (wild > landrace > cultivar; Figure S12e, Supporting Information) and the stepwise allele frequency accumulation of superior alleles of *gcp6*‐*MPC5* reflected domestication advantage by human selection for decreasing grain chalkiness (Figure S12f, Supporting Information). These dynamics domestication features highlight their dual selection history: *GCP6* fine‐tunes environmental responsiveness while *MPC5* stabilizes constitutive grain chalkiness. These findings suggest that the weak‐functional *GCP6* coupled with the functional *MPC5* (*gcp6*‐*MPC5*) is the optimal genotype for improving grain quality with low phenotypic plasticity during rice domestication breeding. Based on the grain shape results of genetic materials, we found that the seed‐setting rates of *GCP6*‐CR and *MPC5*‐OE lines (grain width narrowed and the average grain chalkiness decreased) were decreased at both high and normal temperatures (Figure S13a,b, Supporting Information). However, the seed‐setting rates of *GCP6*‐OE and *MPC5*‐CR lines (grain width became wider and the average grain chalkiness increased) were not different at both high and normal temperatures (Figure S13c,d, Supporting Information). Thus, our results further demonstrate that the weak‐function *gcp6* with the functional *MPC5* (*gcp6*‐*MPC5*) is an excellent genotype combination for enhancing the plasticity of low chalky phenotypes in improving grain quality in rice breeding, offering broad application value.

To confirm the genetic relationship between *MPC5* and *GCP6*, we created the *gcp6 mpc5* double mutants (Figure 7h,m). Compared to the control ZH11, the double mutants exhibited enhanced grain chalkiness (GCR and GCD), similar to the *mpc5* single mutant (Figure 7h–k). The double mutants also exhibited wider grain phenotype, consistent with the *mpc5* single mutant (Figure 7l,m). These findings genetically confirmed that *MPC5* functions downstream of *GCP6*.

Based on our experimental data, we proposed a working model elucidating the trade‐off between phenotypic mean and plasticity of grain chalkiness and width regulated by the GCP6‐*MPC5* module (Figure 7n). Reduced GCP6 expression due to natural variations alleviates this repression on *MPC5*, elevating *MPC5* expression levels which decrease the phenotypic mean of both grain chalkiness and width while enhancing their phenotypic plasticities. Conversely, high GCP6 expression in wild‐type haplotypes suppresses *MPC5*, increasing phenotypic mean but reducing plasticity. Additionally, high temperature independently elevates grain chalkiness and grain width means while diminishing plasticity.

## 3 Discussion

The trade‐off between phenotypic mean and plasticity is a critical factor controlling a trait in crops. A trait with higher phenotypic mean value tends to exhibit greater stability across environments, while a trait with higher plasticity is more sensitive to environmental fluctuations, often at the cost of a reduced phenotypic mean. However, in current diverse and complex environmental contexts, dissecting the mean‐plasticity trade‐off and utilizing its mechanism in crops is still a great challenge. Our comprehensive analysis of five‐year phenotypic data from a rice mini‐core collection provides valuable insights into the global genetic architecture of grain chalkiness, a key determinant of grain quality in cereals. By identifying the comprehensive genetic basis of grain chalkiness plasticity and outlining its complex interaction with environmental factors, we revealed the major genetic and molecular module underlying the trade‐off between phenotypic mean and plasticity in rice. Significant phenotypic plasticity was observed across diverse environments, supported by a high coefficient of variation and the identification of three distinct plasticity parameters (Figure 1). This underscores the adaptive potential of rice to environmental fluctuations and highlights the importance of incorporating plasticity as a key trait in breeding strategies. Furthermore, we observed a significant decrease in grain chalkiness from traditional landraces to modern cultivars, indicating the profound impact of artificial selection on ecological adaptability of rice grain quality. Our multicondition GWAS identified 1587 QTL and 317 hotspot QTL for grain chalkiness (Figure 2), paving the way for targeted breeding initiatives. Additionally, our investigation revealed air temperature and grain width are two major external factors controlling the mean‐plasticity trade‐off and its genetic dissection (Figures 4 and 5). Moreover, we identified two major hotspot QTGs, *MPC5* and *GCP6*, forming a key natural GCP6‐*MPC5* module, that regulate the trade‐off in response to environmental temperatures. We also elucidated the molecular mechanism by which GCP6 inhibits *MPC5* expression, thereby promoting grain chalkiness plasticity synergically (Figures 3, 6, and 7). The GCP6‐*MPC5* regulatory module can be leveraged in breeding program to develop superior‐quality rice suited for a warming climate. Overall, our research advances the understanding of the genetic and plasticity frameworks underlying grain chalkiness in rice, and proposes molecular breeding strategies for enhancing grain quality without compromising essential plasticity traits.

This study revealed, for the first time, distinct genetic foundations underlying both the mean and plasticity of grain chalkiness. Although numerous QTL for grain chalkiness have been identified, most previous studies relied on specific bi‐parental populations, limiting investigations into phenotypic plasticity and overlooking loci present in natural environments. To address this gap, we conducted a global analysis of 533 accessions across five diverse environments. Our results revealed that the grain chalkiness phenotypic mean were generally more dispersed than plasticities, indicative of a major trade‐off effect among grain chalkiness traits (Figure 1). This finding highlights the complex and multifaceted nature of phenotypic expression of grain chalkiness mean. Moreover, our results showed that the heritability and PVEs of phenotypic means and nonlinear plasticities exceeded 50% (Figure 1), showcasing the significant genetic determinants influencing both grain chalkiness mean and its plasticity. Through a global GWAS analysis, we identified 1587 QTL and 317 hotspot QTL distributed across all rice chromosomes (Figure 2). These QTL are controlled by a combination of major and minor QTL as well as environmental variables, revealing the intricate, multifactorial architecture of grain chalkiness (Figure 2; Table S2, Supporting Information). This approach contrasts with traditional experimental designs,^[^ ^22^, ^24^ ^]^ providing a broader understanding of genetic interactions and environmental influences on grain quality. Importantly, the identification of these QTL, their genetic interactions, two major genes, along with their critical minimal SNPs represents a significant advancement for rice molecular breeding of grain quality in diverse agricultural contexts and lays a robust foundation for future basic research. Furthermore, no overlapping gene was found among the phenotypic mean, linear plasticity and nonlinear plasticity for nine traits and their underlying QTL, indicating totally distinct genetic architectures of the three sub‐phenotypes of grain chalkiness in rice (Figure 2b). It is interesting that stable (low plasticity) and high grain chalkiness (inferior quality), or variable and superior quality, often occur together in rice. The minimal marker sets identified in our research explain two thirds of grain chalkiness variation (Figure 2c), indicating the feasibility of improving both the mean and plasticity phenotypes of a complex trait simultaneously. Thus, the integrating both phenotypic mean and plasticity into breeding strategy presents a promising approach to develop environmentally resilient, high‐quality rice varieties that can meet global food demands in a warming future.

We confirmed the important contribution of both air temperature and grain width on the phenotypic stability and genetic detection of grain chalkiness. Previous studies have suggested a correlation between high temperature and grain chalkiness formation.^[^ ^32^ ^]^ Our four‐year study further elucidated the strong positive correlation (coefficients > 0.95) between high temperature and grain chalkiness (Figure 4a–d), establishing high temperature as a major external environmental factor in promoting grain chalkiness. In addition, the negative trends observed in the CVs of grain chalkiness under high temperature (Figure 4e,f) further support this positive correlation. We also found that high temperature significantly reduces the number of grain chalkiness QTL and their PVEs (Figure 4g,h), confirming that high temperature hinders the genetic detection of grain chalkiness. We also explored the novel relationship between grain size and chalkiness formation across various environments (Figure 5), identifying a positive correlation between grain width and chalkiness plasticity. While prior studies have suggested the influence of rice grain shape on chalkiness formation,^[^ ^36^ ^]^ comprehensive researches are limited. Unlike previous studies, we assessed the direct effect of grain width on grain chalkiness plasticity across environments and found the positive correlation between grain width plasticity and grain chalkiness plasticity (Figure 5). Furthermore, the relationships between temperature, chalkiness, and grain size were further validated by the key hotspot‐QTL molecular module, GCP6‐*MPC5*. We analyzed the influence of grain width on grain chalkiness by studying common and specific QTL identified by GWAS in *GW5/gw5* grouped accessions. Our findings showed that slender‐grain genotypes (*GW5*) exhibit more QTL and greater cumulative PVEs compared to *gw5* accessions (Figure 5d,e), suggesting that slender‐grain genotypes are more likely to reveal the genetic basis of grain chalkiness. Considering the decreasing trend of grain chalkiness (Figure 1e) and the prevalence of slender grain shapes, mostly carrying the functional *MPC5/GW5* allele during rice domestication,^[^ ^25^, ^26^, ^37^ ^]^ we speculate that the reduction in grain width may result from human selection of superior appearance cultivars with lower grain chalkiness during *indica* rice improvement. The elucidation of temperature‐chalkiness and grain size‐chalkiness relationships provide valuable insights into the interactions between external environmental factor, internal grain morphological attribute and grain quality.

The interaction between GCP6 and *MPC5* positively regulates grain chalkiness plasticity under natural high temperature in rice. High temperature during the grain‐filling stage significantly reduces seed weight and grain quality, posing a major challenge for breeders and farmers.^[^ ^32^, ^38^ ^]^ Previous studies have linked high temperature to starch imbalance, storage substance content, ROS accumulation, and UPR response, all of which contribute to chalky rice.^[^ ^39^, ^40^, ^41^ ^]^ In this study, we identified two major H‐QTL: H‐QTL144, negatively regulating phenotypic mean and positively regulating phenotypic plasticity, and H‐QTL163, positively regulating phenotypic plasticity. Moreover, two major genes *MPC5*/*GW5*/*GSE5* and *GCP6* were discovered to be responsible for H‐QTL144 and H‐QTL163, respectively. The intricate molecular mechanisms by which the grain‐width master gene *GW5*/*GSE5* influences the phenotypic plasticity of grain chalkiness remain to be fully understood.^[^ ^36^, ^37^, ^39^, ^42^ ^]^ Our findings confirmed an antagonistic or trade‐off effects of *MPC5* (located in the largest‐effect hotspot H‐QTL144) on phenotypic mean and plasticity of grain chalkiness and width using both the mini‐core collection and transgenic lines (Figure 3; Table S3, Supporting Information), reinforcing the dual functional nature of this gene. This trade‐off highlights the challenge of genetic improvement, where selecting high mean values could reduce phenotypic plasticity, and vice versa. Understanding this balance is critical for optimizing grain chalkiness traits under varying environmental conditions. The transcriptional factor *GCP6* is associated with high temperature QTL and linear plasticity QTL, and its mutant showed reduced grain chalkiness and width but increased their variability (Figure 6; Figure S9, Supporting Information), showing a great breeding potential under high temperature. Mechanistically, GCP6 acts as a transcriptional repressor of *MPC5*, demonstrating the trade‐off effects on phenotypic plasticity (Figure 7). GCP6 biochemically and genetically inhibited *MPC5* expression, and is essential for the up‐regulation of *MPC5* under high temperature, because *MPC5* expression does not increase in response to high temperature when *GCP6* was absent (Figure 7). Although GCP6 repressing *MPC5* expression, their interaction enhances the plasticity of chalkiness at high temperature. In brief, we found that a natural key module of GCP6‐*MPC5* controls the mean‐plasticity trade‐off in rice. This also suggests that the interaction of GCP6 and *MPC5* may play a critical role in maintaining the balance between grain yield and quality in high temperature conditions. Therefore, incorporating *GCP6* and *MPC5* into breeding programs is essential for improving rice quality under high temperature.

In conclusion, our study not only enhances the understanding of the trade‐off between phenotypic mean and plasticity of grain chalkiness across different environmental conditions through identifying the GCP6‐*MPC5* regulatory module, but also provides a new proof‐of‐concept strategy to improve grain quality by simultaneously considering both of them.

## 4 Experimental Section

### Phenotypic Data Collection of Grain Chalkiness Traits in Five Environments

A rice min‐core collection of 533 accessions was planted under natural field conditions at the Experimental Stations of Huazhong Agricultural University, Wuhan (2012–2014, 2017) and Hainan (2013), China. Field planting and management refer to the methods of Li et al.^[^ ^11^ ^]^ Paddy rice samples were dehulled using a JLG‐III husker (Sinograin, Zhengzhou, China) to generate brown rice, followed by manual visual assessment of grain chalkiness phenotypes according to the established criteria detailed below. Grain chalkiness in rice, including grain white belly, grain white core and floury endosperm phenotypes, was assessed visually. The percentage of chalky grains in the total number of dehulled grains was used as the measurement of grain chalkiness rate (GCR, %). Grain chalkiness area (GCA, %) is defined as the percentage of chalkiness area to total area of the dehulled rice. Grain chalkiness degree (GCD, %) is defined as the product of grain chalkiness rate by grain chalkiness area. Because of the high temperature of 2013 (Wuhan), the high‐temperature chalkiness phenotype is defined as the difference of the phenotypes in 2013 (Wuhan) with the ones in other four years (Table S1, Supporting Information). GCA is a comprehensive evaluation index for grain chalkiness area, which integrates measurements of WCA (white core area), WBA (white belly area), and FEA (floury endosperm area). GCD is a comprehensive evaluation index for grain chalkiness degree, which integrates measurements of WCD (white core degree), WBD (white belly degree), and FED (floury endosperm degree). GCR is a comprehensive evaluation index for grain chalkiness rate, which integrates measurements of WCR (white core rate), WBR (white belly rate), and FER (floury endosperm rate). The images of grain chalkiness phenotypes were recorded by a scanner EPOSN‐V300.

### Phenotype Variance Decomposition

First, all grain chalkiness phenotypic data were checked for normal distribution by Shapiro‐Wilk test. Because some phenotypic data do not conform to the normal distribution, the linear mixed model was fitted using *lme4* package to assess the effects of different variances and their interaction (i.e., effects of genotype by environment) on all kinds of grain chalkiness phenotypes. The model formula was chalkiness ≈ (1|Line) + (1|Env) + (1|Line:Env). The statistical significance of the random effects was estimated by LRT test using the *ranova()* function of the *lmerTest* package. The heritability was estimated by *H^2^* = *V*_g_/(*V*_g_ + *V*_E_ + *V*_GE_/*m* + *V*_e_/*n* × *m*), where “*m*” represents the number of environments, and “*n*” represents the number of individuals. All statistic tests were performed with the *R* language (version 4.0.0). The PVE of the phenotypic mean, linear plasticity, and nonlinear plasticity for 10 grain chalkiness traits were evaluated using the 4131700 SNP of the 533 accessions by GEMMA.^[^ ^35^, ^43^ ^]^ The proportion of phenotypic variance explained (PVE) by individual genetic variants in Genome‐wide Efficient Mixed Model Association (GEMMA) analyses is calculated using the following standardized formula

(1) $$\mathrm{PVE}=\frac{\beta^{2}}{\beta +\frac{se^{2}\times N}{\mathrm{MAF}\times \left(1-\mathrm{MAF}\right)}}$$

where the parameters are defined as

*β*: Effect size estimate of the genetic variant (regression coefficient from the mixed model); MAF: Minor allele frequency of the variant; *se*: Standard error of the effect size estimate; *N*: Effective sample size calculated as *N* = (*N*_total_ – *N*_missing_), *N*_total_: Number of total individuals analyzed, *N*_missing_: Number of individuals with missing genotype data for the variant.

### Phenotypic Plasticity Decomposition (Stability Analysis)

The phenotypic plasticity of grain chalkiness and width was assessed using a Bayesian Finlay–Wilkinson Regression (FWR) procedure implemented in the FW package according to Kusmec et al.^[^ ^5^ ^]^ Briefly, the FW package jointly estimates the parameters of the genotype‐specific FWR equation

(2) $$y_{ij}=\mu +g_{i}+\left(1+b_{i}\right)h_{j}+\varepsilon_{ij}$$

where *y_ij_* represents the phenotype of the *i*th genotype measured in the *j*th environment, *g_i_* denotes the main effect of the *i*th genotype, *h_j_* signifies the main effect of the *j*th environment, *ɛ_ij_* is an error term assumed to follow an independent and identically distributed normal distribution with a mean of zero and a variance of *σ_ɛ_*,^[^ ^2^ ^]^ and (1 + *b_i_*) represents the change in expected performance of the *i*th genotype per unit change in the environmental effect (*h_j_*). The regression parameters were estimated using *A* = *H* = *I* as described by Kusmec et al. for the confound population structure,^[^ ^5^ ^]^ where *I* is the identity matrix. Values of *g_i_* estimate genotypic mean phenotype values (mean plasticity hereafter). FW returns estimates of *b_i_* and the (1 + *b_i_*) was recorded as a linear response to the environment (linear plasticity hereafter). The variance of *ɛ_ij_* was recorded as a measure of the nonlinearity in that accession's response to the environment. The residual variances were log‐transformed for further analysis (nonlinear plasticity hereafter).

The coefficient of variation (CV) is a statistical measure that expresses the ratio of the standard deviation to the mean, providing a standardized way to compare the degree of variation between datasets with different units or means. It is calculated using the following formula

(3) $$\mathrm{CV}=\left(\sigma /\mu \right)\times 100\%$$

where *𝜎* represents the standard deviation of the dataset, and 𝜇 represents the mean of the dataset. The resulting CV is expressed as a percentage, allowing for easier comparison of variability across different contexts. A higher CV indicates greater relative variability, while a lower CV suggests more consistency in the data.

### Phenotypic Plasticity in Response to the Environment (PPRE) Model

We employed the PPRE model^[^ ^4^ ^]^ to evaluate the effect of the environmental factor on grain chalkiness plasticity (Figure 4i). Here, the environmental factor includes temperature at the filling stage in 12_WH, 13WH, and 14_WH, as well as the grain width values in 12_WH, 13_HN, 13WH, and 14_WH. In the PPRE model, a linear regression analysis was performed using the ten phenotypes of each individual in each environment as the response variables and the environmental index values as the explanatory variables. In addition to considering the intercept and slope, the mean *R* ^2^ of individuals was particularly focus on to evaluate the contribution of the environmental factor to the grain chalkiness plasticity.

### Pleiotropic Effect of Grain Width on Grain Chalkiness

To validate the impact of grain width on grain chalkiness, we investigated the Pearson correlation between grain width value of hulled grains and each grain chalkiness phenotype in the natural population using the *cor.test()* of *R*. The effect was examined of the major gene *GSE5/GW5* of grain width on grain chalkiness by Wilcoxon rank sum test. The contribution of the phenotypic plasticity of grain width was further evaluated on the phenotypic plasticity of grain chalkiness through linear regression using the *lm()* function in *R*.

### Genome‐Wide Association Studies of Five‐Category Phenotypes of Grain Chalkiness in Rice

Considering factors affecting grain chalkiness formation, we primarily focused on five categories of phenotypes to perform GWAS analysis. (1) Phenotypic mean, a phenotype generated by FWR analysis that indicates the primary genotype effect. (2) Plasticity phenotypes, including linear and nonlinear plasticity phenotypes, also generated by FWR analysis, presenting the environmental effects. (3) Environment‐specific phenotypes, referring to the raw grain chalkiness phenotype of an individual in a given year/place. (4) Condition‐specific phenotypes, referring to the phenotype of individuals grouped by the potential factors that might influence grain chalkiness formation such as *indica*/*japonica*, *W*x and grain‐width genes *GSE5*/*GW5*. (5) Grain shape phenotypes which have pleiotropic effect on grain chalkiness, such as grain length, width, length/width ratio and thickness (Table S1, Supporting Information).

For genome‐wide association studies (GWAS), a sequenced diversity panel consisting of 533 accessions was used. SNPs with a minor allele frequency (MAF) below 5% and a missing rate below 10% were filtered out, leaving 4131700 SNPs of 529 accessions for the analysis. Factored spectrally transformed linear mixed models (FaST‐LMM) was adopted to perform GWAS^[^ ^44^ ^]^ due to its advantages in computational speed and memory usage. The Manhattan plots were drawn by the *qqman* package. The threshold for genome‐wide significance was determined by Bonferroni correction (that is, corrected *P* = 0.05/*n*, in which *n* is the number of independent SNPs across the genome). The *n* was estimated by pruning the SNP dataset using PLINK (version 1.9) with arguments ‘–indep‐pairwise 1000 kb 10 0.3′, retaining 36508 independent SNPs.^[^ ^45^ ^]^ Here, the genome‐wide significance level was set at 1.37 × 10^−6^ using the Bonferroni method. Linkage‐disequilibrium block analysis was performed by a *R* package LDheatmap.^[^ ^46^ ^]^ The heritability or PVE of each trait of each environment was estimated by GEMMA using the genome‐wide SNPs.^[^ ^43^ ^]^

### Genetic Validation of Candidate Genes for Grain Chalkiness in Rice

For the CRISPR/Cas9‐*GCP6* vector construction, two gene‐specific guide sequences (sgRNAs) were designed and integrated into the OsU6 promoter using overlapping PCR with *GCP6*U6‐F and *GCP6*U6‐R primers. Then the sgRNA transcriptional unit was inserted into pCXUN‐CAS9 plasmid at the *Kpn* I site using the ClonExpress II One Step Cloning Kit (Vazyme, China) to create CRISPR/Cas9‐*GCP6* plasmid.

To create overexpression plants, the coding sequence (CDS) of *GCP6* from ZH11 (H2 haplotype) was amplified using *GCP6*‐OE‐F and *GCP6*‐OE‐R primers, and then cloned into to the PU2301‐Flag vector with the ClonExpress II One Step Cloning Kit (Vazyme, China) to generate *GCP6* overexpression plasmid.

For *GCP6*‐complementation plants, the H2 haplotype was introduced into the H1 haplotype rice variety. For this purpose, the 3254‐bp genomic sequence was amplified of *GCP6* from the ZH11 (H2 haplotype) using the primers *GCP6‐*Com‐F and *GCP6‐*Com‐R. For *MPC5*‐complementation plants, the 7897‐bp genomic sequence of *MPC5* was amplified from 9311 using the primers *MPC5‐*Com‐F and *MPC5‐*Com‐R. Subsequently, the amplified sequences were cloned into the pCAMBIA 1301‐Flag vector using the ClonExpress II One Step Cloning Kit (Vazyme, China) to generate the complementation plasmids, separately.

All the plasmids were transformed into the *Agrobacterium tumefaciens* strain EHA105. Subsequently, the CRISPR/Cas9‐*GCP6* and *GCP6*‐OE plasmids were introduced into ZH11, the *GCP6*‐complementation plasmid was introduced into Fengaizhan (FAZ), and the *MPC5*‐complementation plasmid was introduced into SLG by *Agrobacterium*‐mediated transformation, respectively.^[^ ^34^ ^]^ The relevant PCR primer sequences are provided in Table S6 in the Supporting Information.

### RNA Extraction and qRT‐PCR

Total RNA was extracted using TRIzol (TransGen Biotech, BJ, CHN) and stored at −80 °C. Subsequently, 2 µg of RNA was reversely transcribed using ABScript II cDNA First‐Strand Synthesis Kit (Abclonal, WHU, CHN) according to the manufacturer's instructions. Primers for qRT‐PCR were designed using Snapgene software and their specificity were checked through NCBI primer‐blast; these primers can be found in Table S6 in the Supporting Information. The qRT‐PCR reaction mixture, with a total volume of 10 µL, consisted of 1 µL of cDNA, 0.2 µL each of reverse and forward primers, 2 µL of double‐distilled water, and 5 µL of Hieff UNICON Universal Blue qPCR SYBR Green Master Mix (Yeasen, SHH, CHN). The Ubiquitin gene (LOC_Os03g13170) was used as internal controls. qRT‐PCR was performed using the QuantStudio7 (Thermo, MA, USA). Each reaction was run with four technical replicates, and all the relative expression levels were determined by the 2^−ΔΔ^ *^Ct^* method.

### Transactivation Activity Analysis

The full‐length CDSs of *GCP6* was amplified and cloned into the vector pCAMBIA 1300‐Flag to generate the effector. The promoter of *MPC5* (1045 bp upstream of ATG) from ZH11 or 9311 was amplified and cloned into the vector pGreenII 0800‐LUC to generate a reporter. The plasmids were transferred into the A. tumefaciens strain GV3101 by electroporation, and the Agrobacterium carrying the reporter together with the effector were coinfiltrated into *N. benthamiana* leaves. Following the manufacturer's instructions, Firefly and Renilla luciferase activities were measured after coinfiltrated for 48 h using a Dual‐Luciferase Reporter Assay System (Promega, Madison, WI, USA) on an automated multimode microplate reader (TECAN Spark). Empty and GCP6‐Flag vectors were used as control. The primers were listed in Table S6 in the Supporting Information.

### ChIP‐qPCR Assay

ChIP assays were performed following the published protocols.^[^ ^47^ ^]^ The rice seed samples for the ChIP assays were collected from transgenic complementation lines of *GCP6*‐Flag. The samples were harvested, fixed in 1% formaldehyde under vacuum for 15 min, and then neutralized with 0.125 m Glycine. Chromatin was isolated from ≈2 g samples of the developing seeds, fragmented to 200–500‐bp fragments by sonication (Bioruptor, BE). Subsequently, GCP6‐Flag protein/DNA complexes were coated with Anti‐Flag antibodies (Abclonal, WHU, CHN) for overnight at 4 °C (IgG group with no Anti‐Flag antibodies). Then the chromatin solution was incubated with Anti‐Flag magnetic beads (Selleck, USA) at 4 °C for more than 6 h for immunoprecipitation. The precipitated DNA was purified and the enriched DNA fragments were determined using qRT‐PCR. All reactions were repeated three times, and the relative enrichment levels were determined by the 2^−ΔΔ^ *^Ct^* method. The primer sequences were listed in Table S6 in the Supporting Information.

### Electrophoretic Mobility Shift Assay (EMSA)

The coding region of *GCP6* was cloned into the pGEX4T‐1(GST‐tag) vector using the *Eco*R I and *Xho* I restriction enzymes (Thermo, MA, USA). The recombinant GST‐GCP6 protein was induced by 1 mM IPTG and expressed in *E. coli* strain BL21. Purification of the GST‐GCP6 protein was carried out using GST Bestarose 4FF resin (Bestchrom, SHH, CHN). The oligonucleotide probe of binding element on the MPC5 promoter was biotin‐labelled at the 5′ end, while the competitor probe remained unlabeled. The probes were synthesized and labelled by the Shanghai Sangon Company (Table S6, Supporting Information). In the biotin‐labeled probe reaction, contained 5 µg of protein and 200 µmol of oligonucleotides were utilized, while the competitor probe reaction contained 5 µg of protein and 6 or 120 × 10^−3^ m oligonucleotides. The oligonucleotides were then incubated with the GST‐GCP6 fusion protein in LightShift Chemiluminescent EMSA Kit (Thermo Fisher Scientific, USA) mixtures at 25 °C for 30 min, following the manufacturer's instructions. The GST‐empty vector protein served as the negative control. Biotin‐labeled probes were visualized using Tanon‐5200 Chemiluminescent Imaging System (Tanon, SHH, CHN).

### Epistasis Analysis of Grain Chalkiness‐Related Genes

First, a dada set was constructed containing 974 grain chalkiness‐related genes manually (Table S7, Supporting Information). The data set contains candidate genes identified in this study, genes reported for chalkiness‐related phenotypes and that from the KnetMiner dataset (https://knetminer.com/) with the keyword “chalkiness”. In order to clarify the genetic interaction between key candidate genes identified in this study and other related genes, we analyzed the pair‐wise epistasis of the data set by a functional regress model that was implemented by FRGEpistasis.^[^ ^48^ ^]^ The combination of rigorous stratification correction, scalable computation, and functional prioritization establishes a robust framework for epistasis mapping without requiring specialized breeding populations. The interactions were only accepted that passed through the threshold of FDR < 0.05. The software can analyze the interactions of genome regions on both same or different chromosomes. Here, we defined all SNPs annotated to a gene as a genomic region. The 10 grain chalkiness traits in the environment with the highest heritability were used as the phenotype input to perform epistasis analyses. The interaction network was visualized by *cytoscape*.^[^^]^

### Marker Selection and Prediction Model for Molecular Breeding

Least Absolute Shrinkage and Selection Operator (LASSO) regression was highly effective in reducing the dimensionality of SNP data. To obtain the minimal markers and the corresponding prediction model, LASSO regression was adopted to select relevant SNPs associated with the chalkiness phenotype with R package *glmnet*. For each of the GCD^Mean^, GCD^LP^, GCA^Mean^, GCA^LP^, GCR^Mean^ and GCR^LP^, independent LASSO regression was performed using the 9581 SNPs of the 974 chalkiness‐related genes. LASSO automatically performs feature selection by shrinking the coefficients of less relevant SNPs to zero. The tenfold cross validation was performed in the feature selection process. The SNPs with nonzero coefficients were identified as potential predictors of the chalkiness phenotype. Then, backward stepwise regression was conducted using stepAIC function of *MASS* package to further prune the SNP number. To evaluate the prediction model, various indicators such as the accuracy, precision etc. can be calculated by *caret* package. The PVE of each model was estimated by linear regression using the selected SNPs.

### Statistical Analysis

Data normality was evaluated by Shapiro–Wilk test prior to each analysis. For normally distributed data, parametric tests were applied (student's *t*‐test or Tukey–Kramer multiple comparisons test). Sample sizes are explicitly specified in the corresponding figure legends. The selected statistical test for each analysis is listed in the corresponding figure legends. Analyses were performed in R v4.1.2.

### Data Availability

The data supporting the findings of this work are available within the paper and its Supplementary Information files. A reporting summary for this article is available as a Supplementary Information file. The SNP data of the mini core collection can be retrieved from http://ricevarmap.ncpgr.cn/download/. Accession codes of all genes or alleles reported in the study are available in GenBank: cDNA of *MPC5*, CK042657.1 [https://www.ncbi.nlm.nih.gov/nucleotide/CK042657.1]; cDNA of *GCP6*, XM_01 578 6619.2 [https://www.ncbi.nlm.nih.gov/nucleotide/XM_015786619.2]. Source data are provided with this paper.

## Conflict of Interest

The authors declare no conflict of interest.

## Author Contributions

J. Z., Y.D., and P.X. contributed equally to the work. J.Z. performed parts of the research, analyzed data and wrote the original draft. Y.D. generated the CRISPR and complementation genetic material of *GCP6* verified its regulatory relationship with *MPC5*, and phenotyped all transgenic lines in multi‐years. P.X. provided the original five‐year grain‐chalkiness phenotype data of the 533 rice germplasms. L.Z., Z.L., and W.Z. participated in the function validation of some candidate genes. Y.L. constructed the complementation material of *GW5* in SLG genetic background. B.C. provided the temperature data of different environments. X.C. provided some help on the qRT‐PCR analyses of *GCP6* and *MPC5* in MH63. Y.F. and Y.L. also assisted with conducting experiments on *GCP6*. Y.L. designed, conceptualized, supervised the research work and wrote the manuscript.

## Supporting information



Supporting Information

Supplemental Tables

## Data Availability

The data that support the findings of this study are available in the supplementary material of this article.
