# Supplementary material for: A Natural Major Module Confers the Trade‐Off between Phenotypic Mean and Plasticity of Grain Chalkiness in Rice
Source: Adv Sci (Weinh). 2025 Aug 22;12(42):e06242. doi: 10.1002/advs.202506242 (PMC12622540; doi:10.1002/advs.202506242)
Supplement: Supplementary file 1 — Supporting Information [file ADVS-12-e06242-s002.docx]

## Supplementary Figures


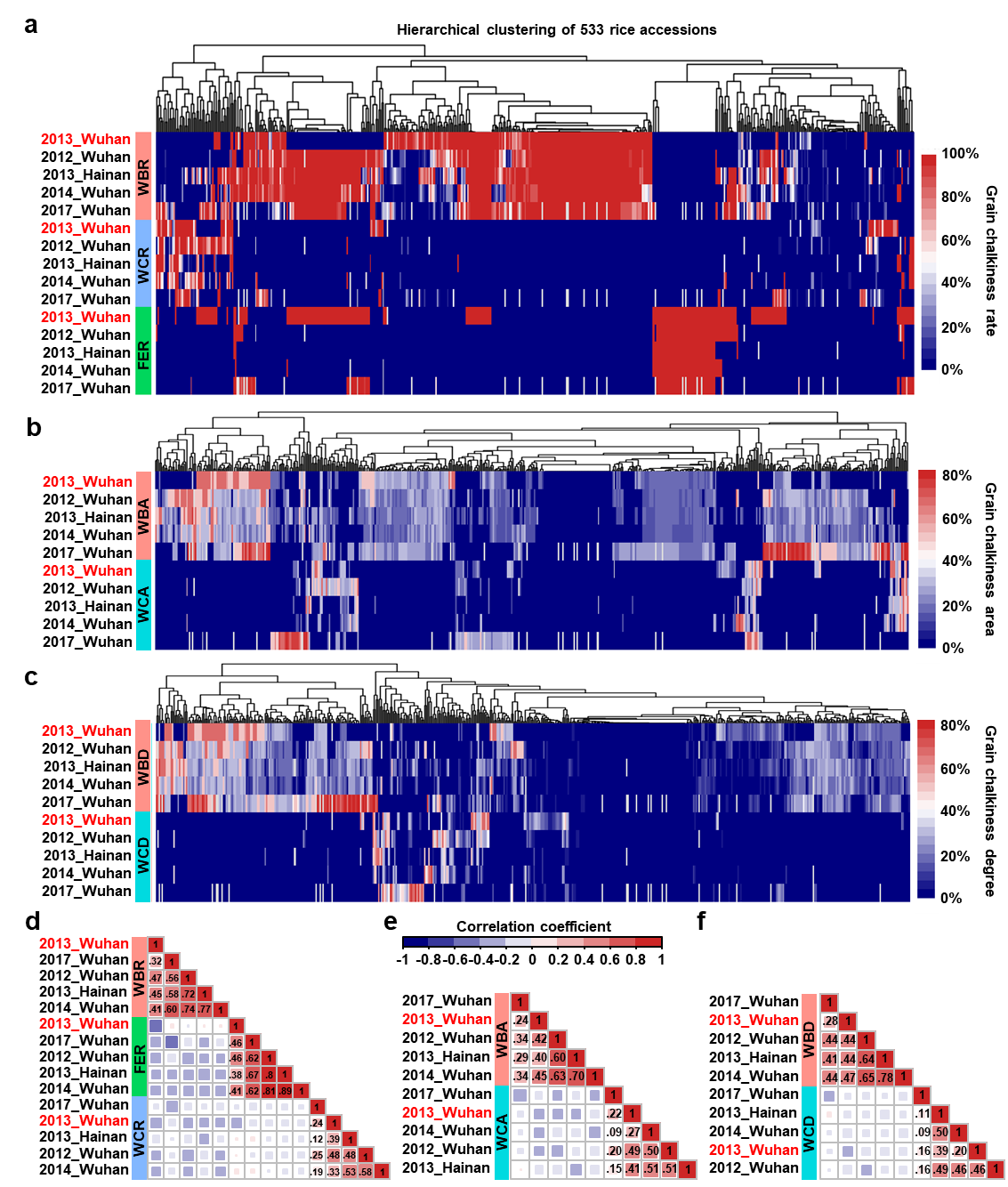


**Figure S1 Natural variation of rice grain chalkiness in a mini-core collection of 533 accessions under five-year environments.** **a–c**, The natural variation of grain chalkiness rate (**a**), grain chalkiness area (**b**) and grain chalkiness degree (**c**) of 533 accessions under five environments. “2013_Wuhan” are highlighted in red for that 2013 is a year with extreme high temperature at rice grain filling stage and the phenotypes are significantly different from other years. **d–f**, The correlations of grain chalkiness rate (**d**), grain chalkiness area (**e**) and grain chalkiness degree (**f**) under five-year environments. WBR, grain white belly rate; WCR, grain white core rate; WBA, grain white belly area; WCA, grain white core area; WBD, grain white belly degree; WCD, grain white core degree; FER, floury endosperm rate.


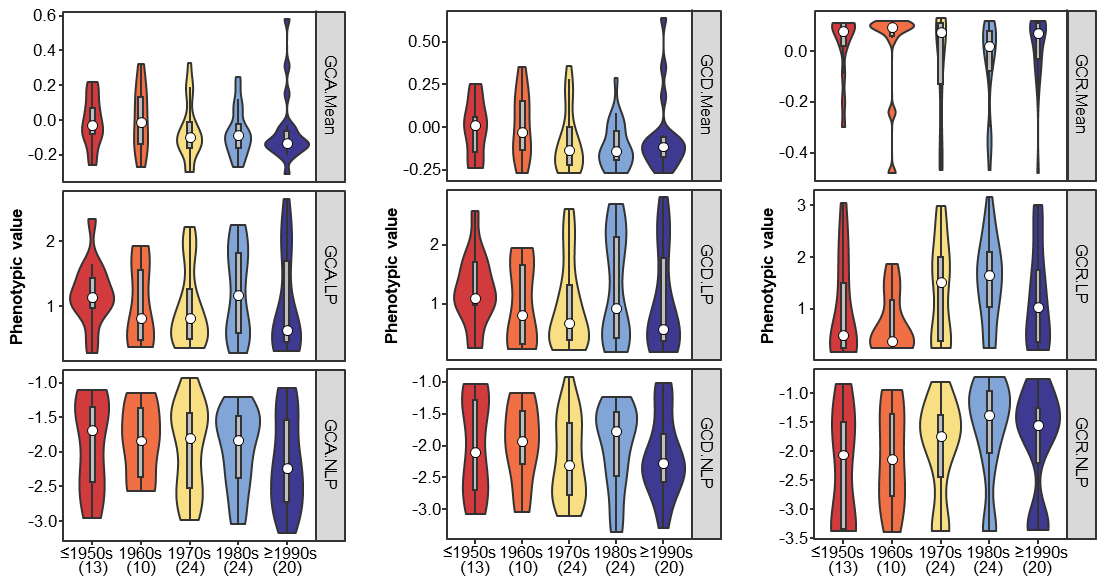


**Figure S2 The variation trends of grain chalkiness phenotypic plasticity from ≤1950s to ≥1990s.** The number in parentheses represents the number of varieties (*n*).


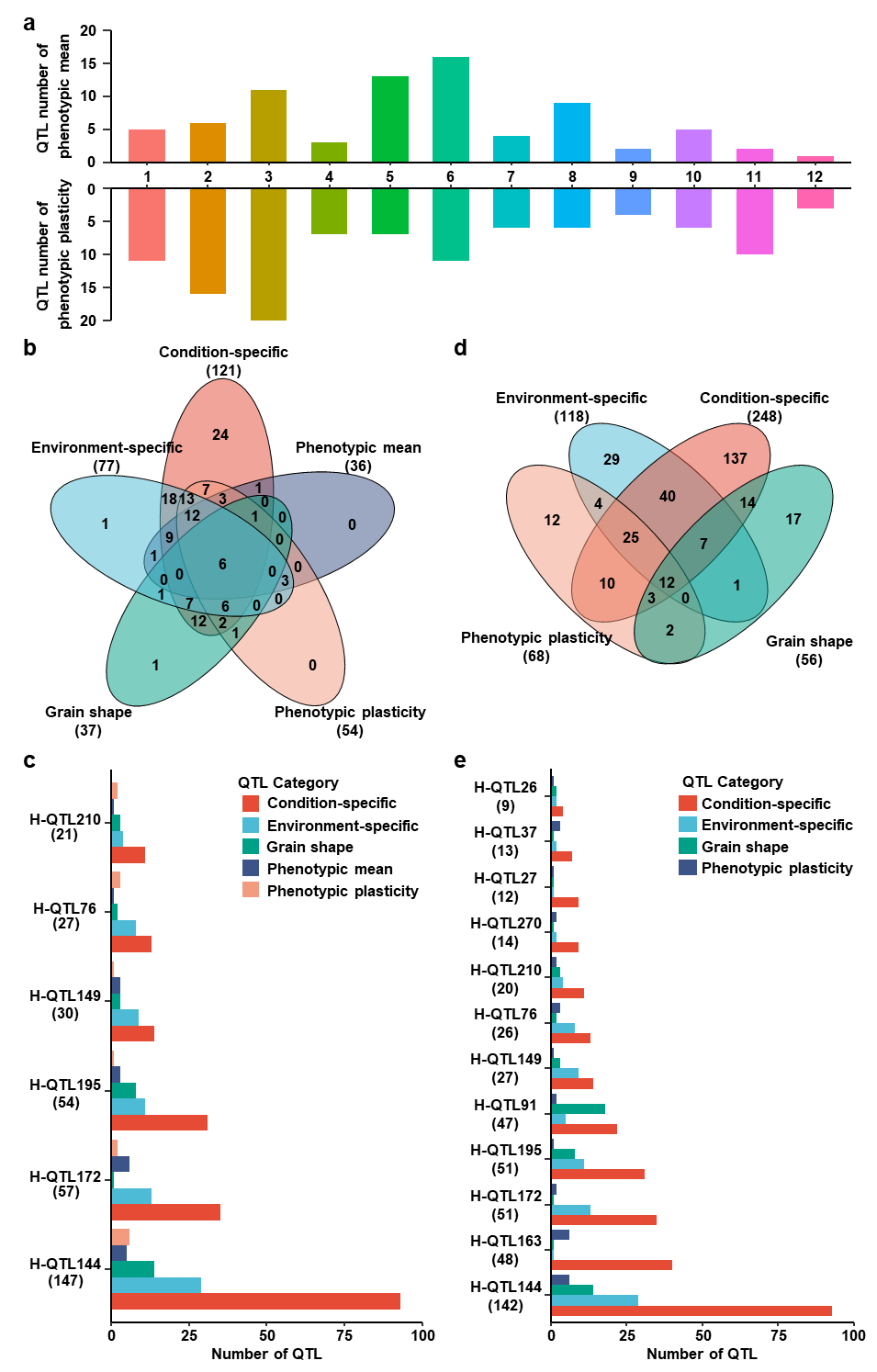


**Figure S3 QTL chromosomal distribution and comparison of H-QTL between the mean phenotype and plasticity phenotype of grain chalkiness.** **a**, The asymmetric chromosomal distributions of QTL for phenotypic mean and plasticity. **b**, Comparisons of H-QTL for the five QTL categories. **c**, The number of QTL of the five categories within the six common H-QTL in **b**. **d**, Comparisons of H-QTL for the four QTL categories mainly for phenotypic plasticity. **e**, The number of QTL of the four categories within the 12 common H-QTL in **c**.


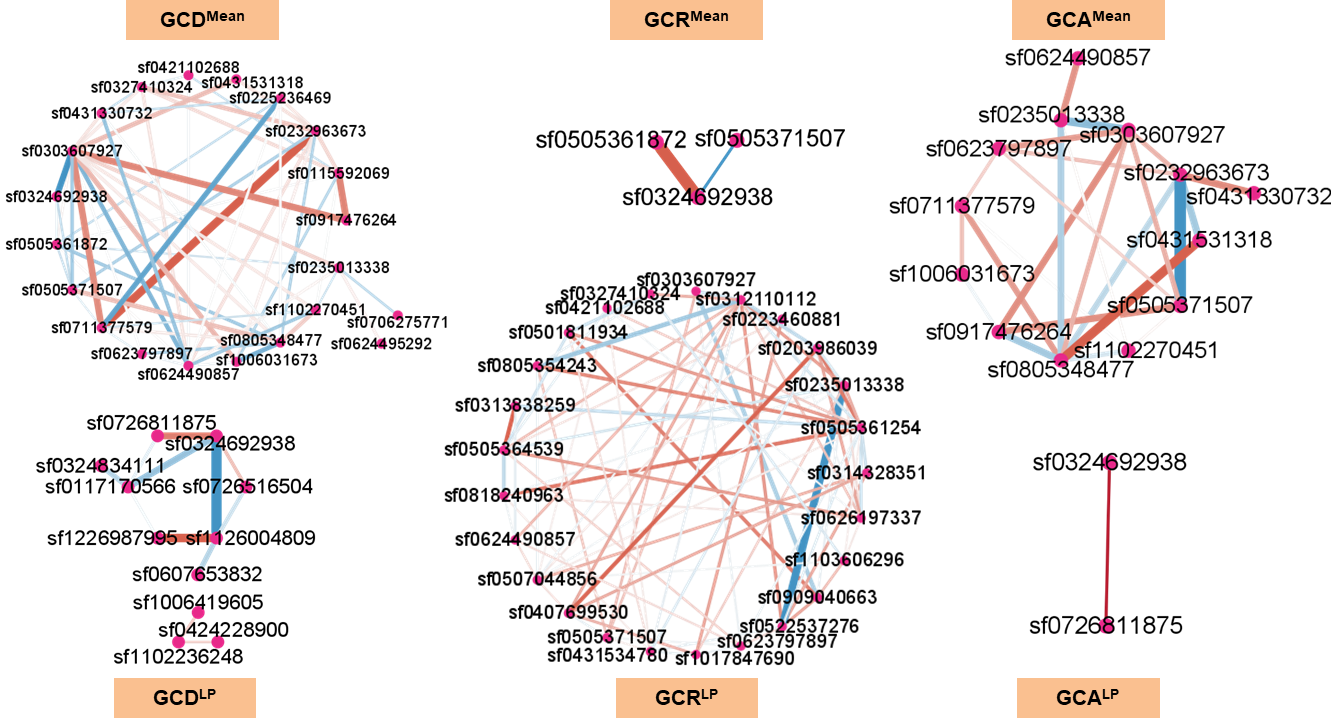


**Figure S4 Genetic interactions of the minimal SNP datasets regulating grain chalkiness mean and plasticity.** The superscripts of Mean and LP represent the phenotypic mean and linear-plasticity of GCD, GCR and GCA, respectively. Blue and brown indicate the negative and positive effect to the phenotype, respectively. The width of the edge indicates the size of the interaction effect. Detailed information is listed in **Table S4**.


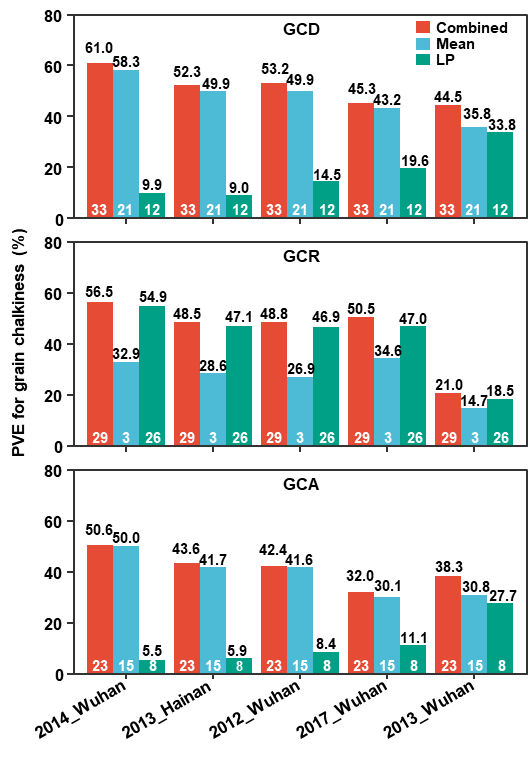


**Figure S5 Phenotypic variance explained (PVE) by SNPs associated with the prediction of mean and plasticity (LP) phenotypes of grain chalkiness, along with their combined SNP sets across five growing seasons.** The five environments on the *x*-axis were arranged in ascending order based on the average temperature during grain filling stage. The values at the top (black) and bottom (white) of the bars indicate the PVE and the number of SNPs, respectively. The SNPs used here were same as that in **Fig. 2c**.


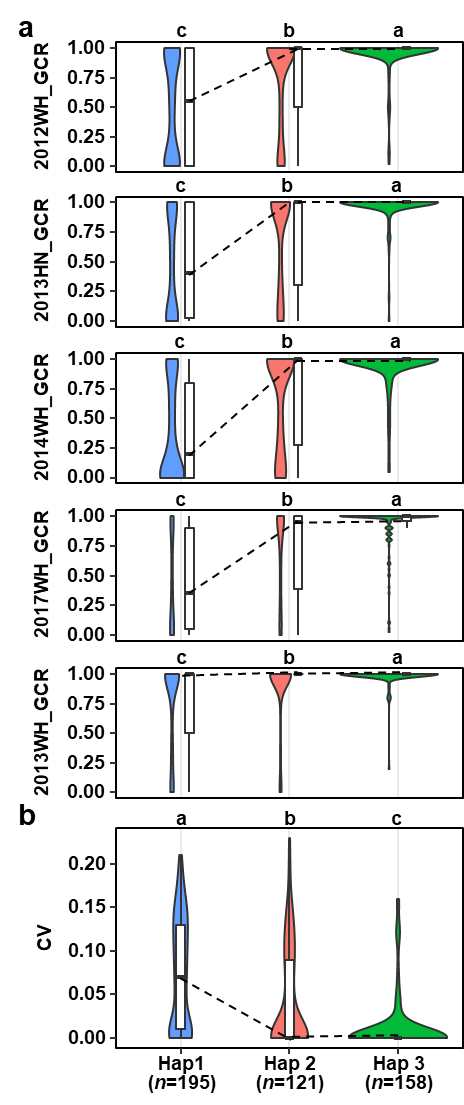


**Figure S6 Environment-specific GCR phenotypes (a) and its CVs (b) of the three haplotypes of *MPC5* in the mini-core collection of 533 accessions.** Letters above boxes​ denote significant group differences (*P* < 0.05) based on ​LSD multiple comparison tests; groups labeled with distinct letters differ significantly.
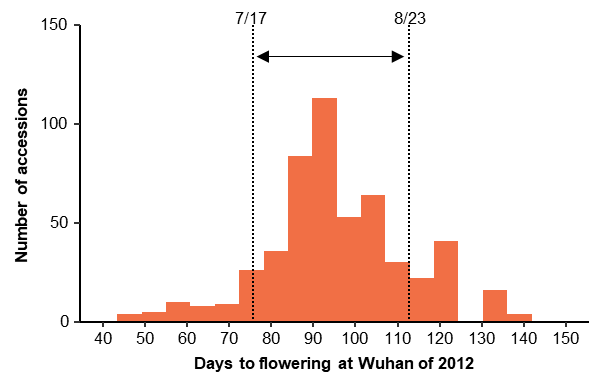


**Figure S7 The distribution of days-to-flowering (DTF) of the accessions in our association panel and the temperature window adopted in this study.**


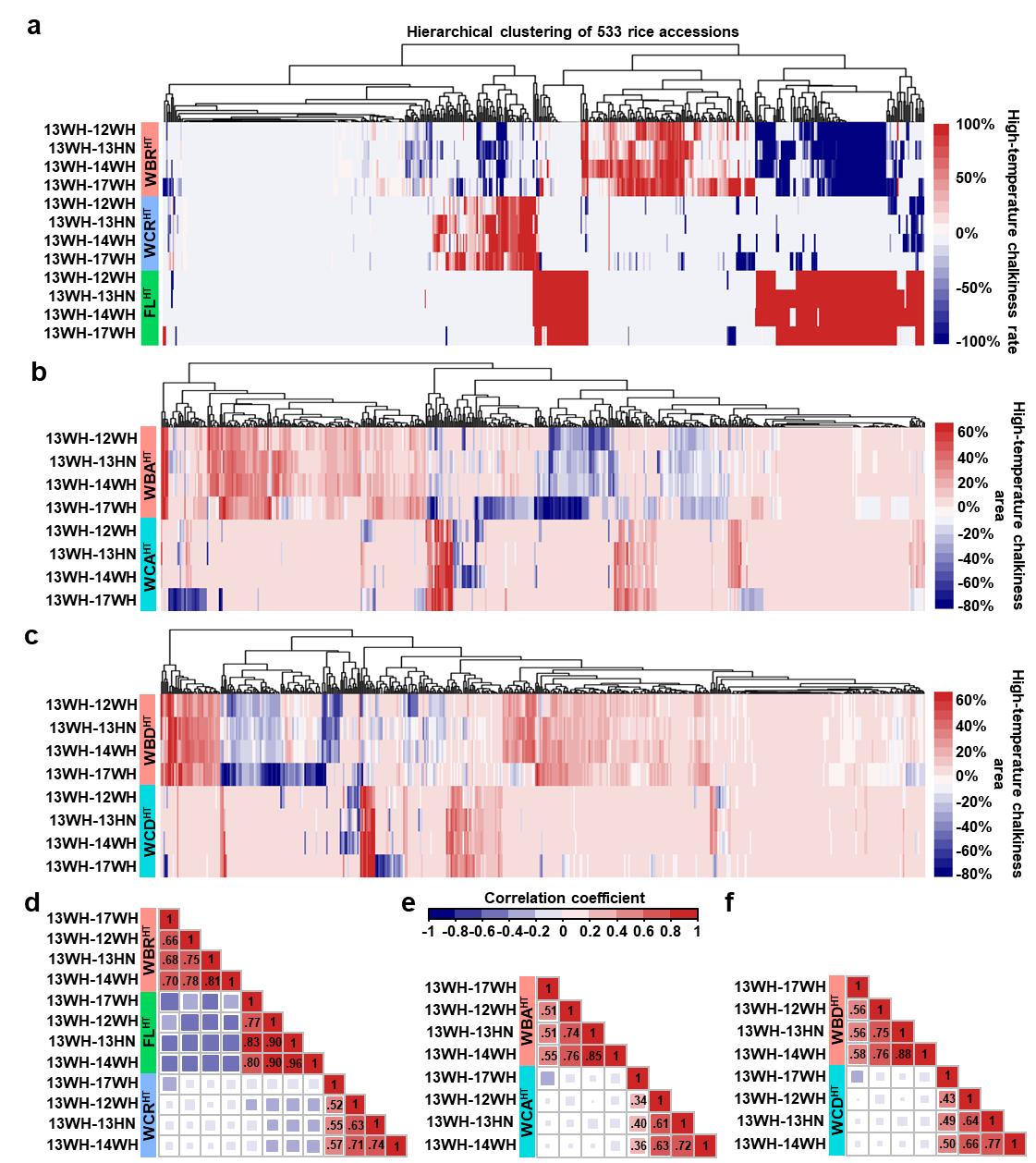


**Figure S8 Natural variation of the DIFF score in the mini-core collection of 533 accessions under five-year environments.** **a–c**, The natural variation of the DIFF of grain chalkiness rate (**a**), grain chalkiness area (**b**) and grain chalkiness degree (**c**) of 533 accessions under five environments. “2013_Wuhan” are highlighted in red for that 2013 is a year with extreme high temperature at rice grain filling stage and the phenotypes are significantly different from other years. **d–f**, The correlations of grain chalkiness rate (**d**), grain chalkiness area (**e**) and grain chalkiness degree (**f**) under five-year environments. WBR^HT^, grain white belly rate under high temperature; WCR^HT^, grain white core rate under high temperature; WBA^HT^, grain white belly area under high temperature; WCA^HT^, grain white core area under high temperature; WBD^HT^, grain white belly degree under high temperature; WCD^HT^, grain white core degree under high temperature; FER^HT^, floury endosperm rate under high temperature.


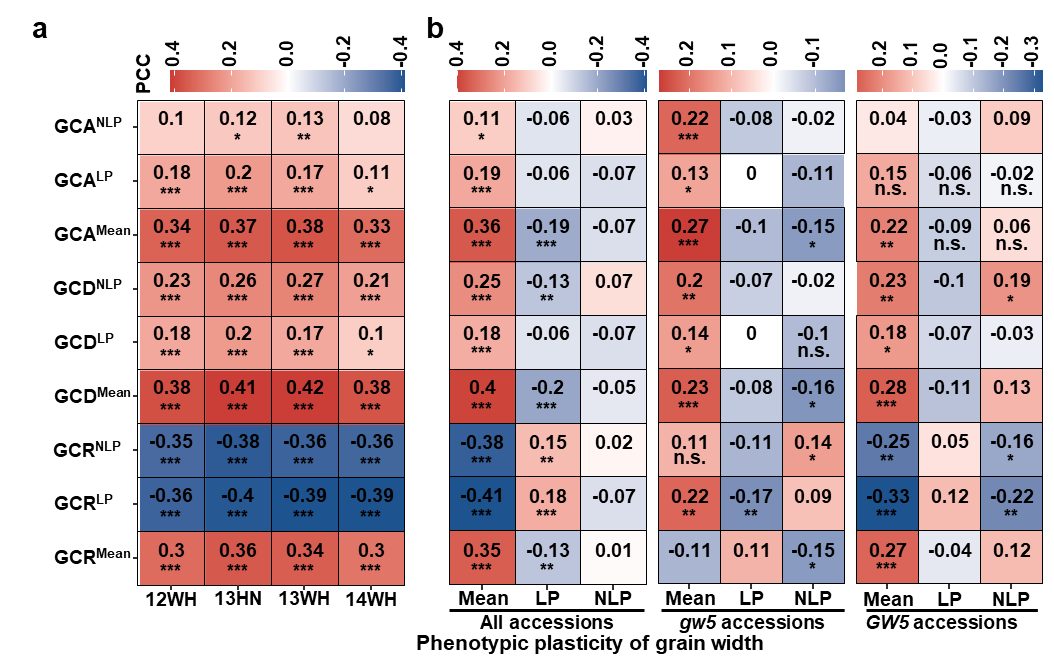


**Figure S9** **Correlation between grain width and chalkiness plasticity.** **a,** Pearson correlation coefficient (PCC) of grain width and grain chalkiness plasticity in the mini core collection of 533 accessions. **b,** PCC of grain-width phenotypic plasticity and grain-chalkiness phenotypic plasticity in all (left panel), *gw5* (middle panel) and *GW5* (right panel) accessions. The color of the cells was scaled for the PCC, while the size of the cells was scaled for the absolute value of the PCC. *P* values indicate the statistical significance of the two-tailed student’s *t*-test. *, *P* < 0.05; **, *P* < 0.01; ***, *P* < 0.001.


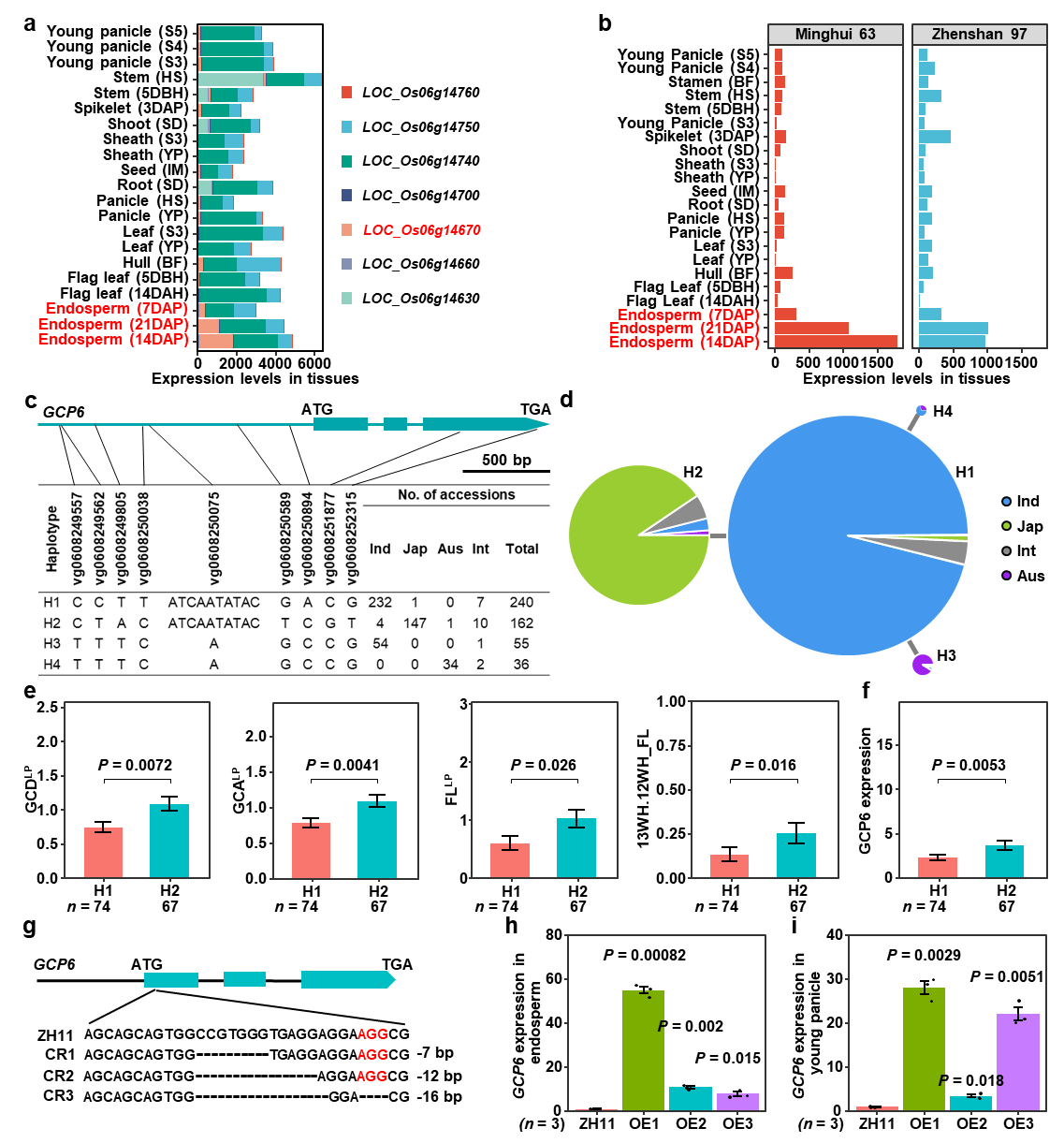


**Figure S10** **The identification of *GCP6* under five-year field trials with diverse natural temperature conditions.** **a,** The expression levels of seven of 12 genes underlying the 98-kb region in tissues of rice cultivar Minghui63. **b,** Spatiotemporal expression pattern of *LOC_Os06g14670* (*GCP6*) in varieties of Minghui63 and Zhenshan97.**c,** Natural variation and haplotype analyses of *GCP6* in 533 rice accessions. **d,** The subspecies differentiation of the haplotypes of *GCP6* by haplotype network analysis. Ind, Jap, Aus, and Int represent *indica*, *japonica*, *aus*, and *intermediate* accessions, respectively. The phenotype plasticity (**e**) and expression level (**f**) of two major haplotypes of *GCP6*. *n* is the number of accessions or the number of individuals of each transgenic line. **g**, The CRISPR/Cas9 target sites of three independent knockout lines for *GCP6*. **h–i**, *GCP6* expression in 5-DAP endosperms (**h**) and 4-cm young panicles (**i**) of over-expression lines. The control was the negative over-expression lines of ZH11 background. Three biological replicates were analyzed for each line (*n* = 3). All data are shown as mean value ± SEM. All the *P* values were produced by two-tailed Student's *t*-tests. SEM, standard error of the mean.

**
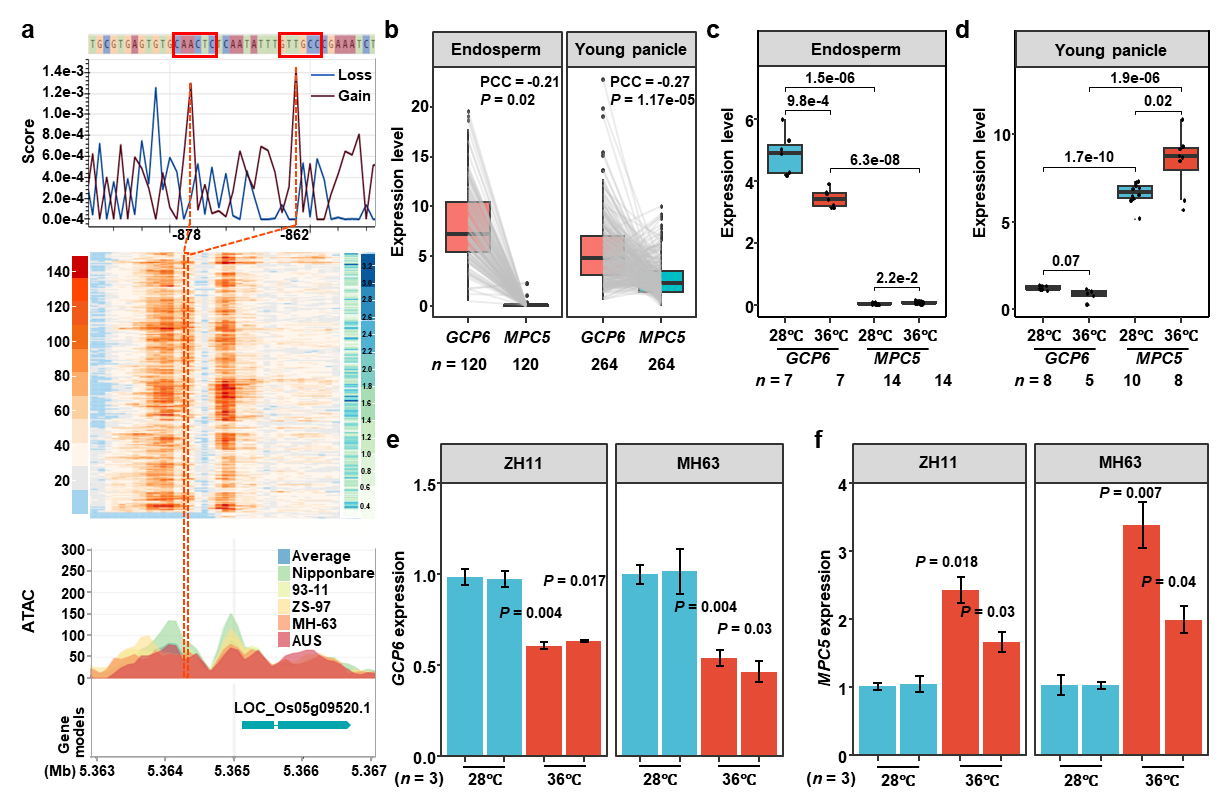
**

**Figure S11 Chromatin accessibility of *MPC5* and co-expression patterns of *GCP6* and *MPC5* from 28°C to 36°C.**

**a,** Chromatin accessibility analyses by ATAC (bottom) and deep learning models (top) revealed the two motifs that are localized in chromatin accessibility peak regions of *MPC5* promoter. “Loss” and “gain”represent reduced and increased chromatin accessibility, respectively. **b**, Co-expression of *GCP6* and *MPC5* in endosperms and young panicles of the mini core collection. **c, d,** Down- and up-regulation of *GCP6* and *MPC5* from 28°C to 36°C in 5-DAP endosperms (**c**) and 4-cm young panicles (**d**) measured by RNA-Seq of the mini core collection, respectively. **e**, Down-regulation of *GCP6* from 28°C to 36°C measured in 5-DAP endosperms of ZH11 and MH63 by qRT-PCR. **f**, Up-regulation of *MPC5* from 28°C to 36°C measured in 5-DAP endosperms of ZH11 and MH63 by qRT-PCR. *P* values indicate the statistical significance of the two-tailed student’s *t*-test. Three biological replicates were used for the assay (*n* = 3).


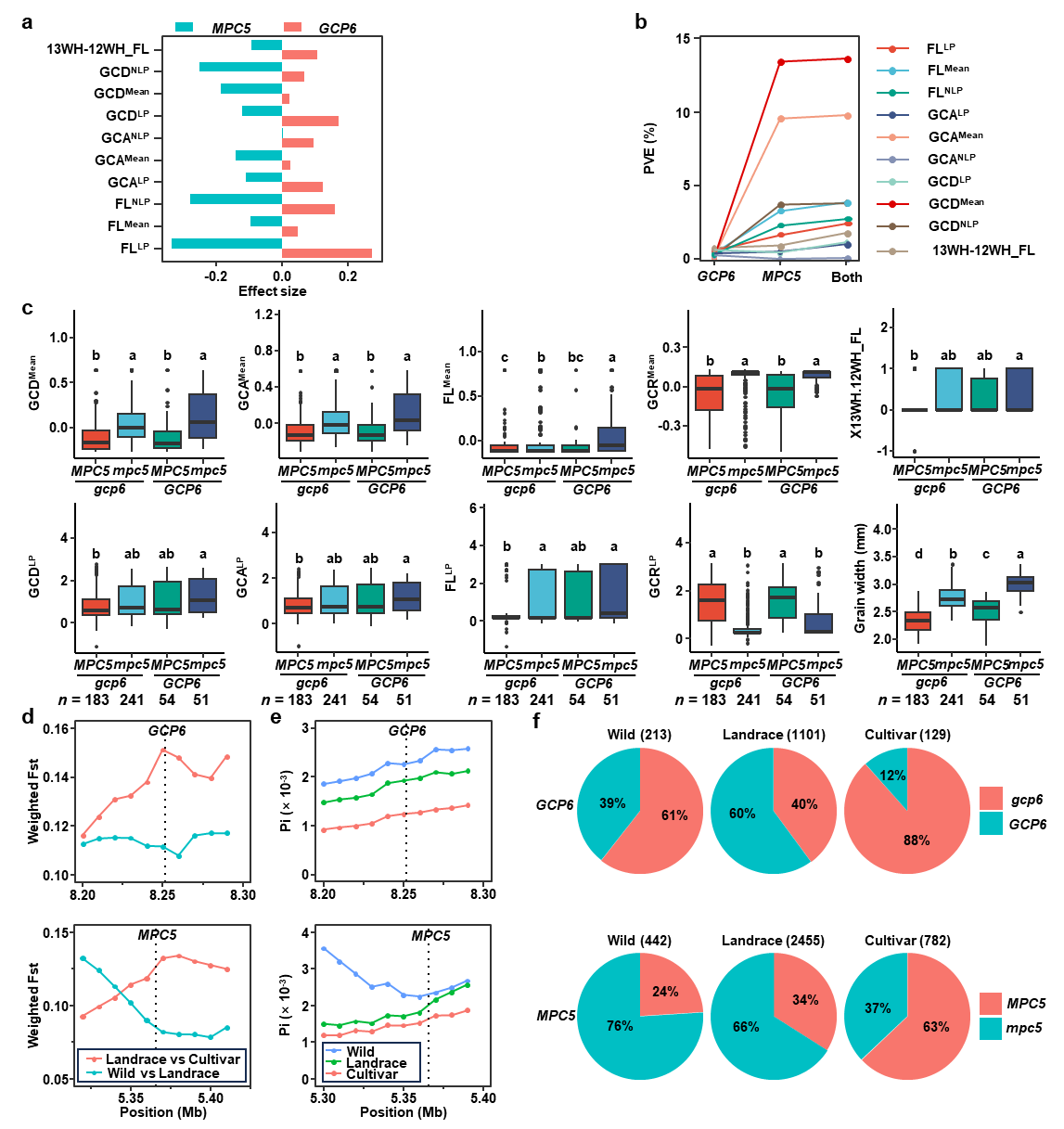


**Figure S12 The effect size of *GCP6* and *MPC5* and grain chalkiness plasticity phenotypes of their different haplotype combinations in the mini-core collection.** **a**, Opposite effects of *GCP6* and *MPC5* on the phenotype plasticity of grain chalkiness by regression evaluation in the mini-core collection. **b**, The two genes together own more PVEs than single gene for ten grain chalkiness plasticity traits. **c**, Phenotype plasticity of grain chalkiness and width of the four haplotypes combining *GCP6* and *MPC5* in the mini-core collection. Different letters above boxes denote significant differences (*P* < 0.05) determined by LSD multiple comparison test. Boxes sharing a common letter are not significantly different. Sample numbers are given in each figure. **d**, Population differentiation index (Fst) of *GCP6* and *MPC5* between wild-landrace and landrace-cultivar. **e**, Genetic diversity (Pi) of *GCP6* and *MPC5* in wild, landrace and cultivars. **f**, Allelic frequency of *GCP6* and *MPC5* in wild, landrace and cultivars.


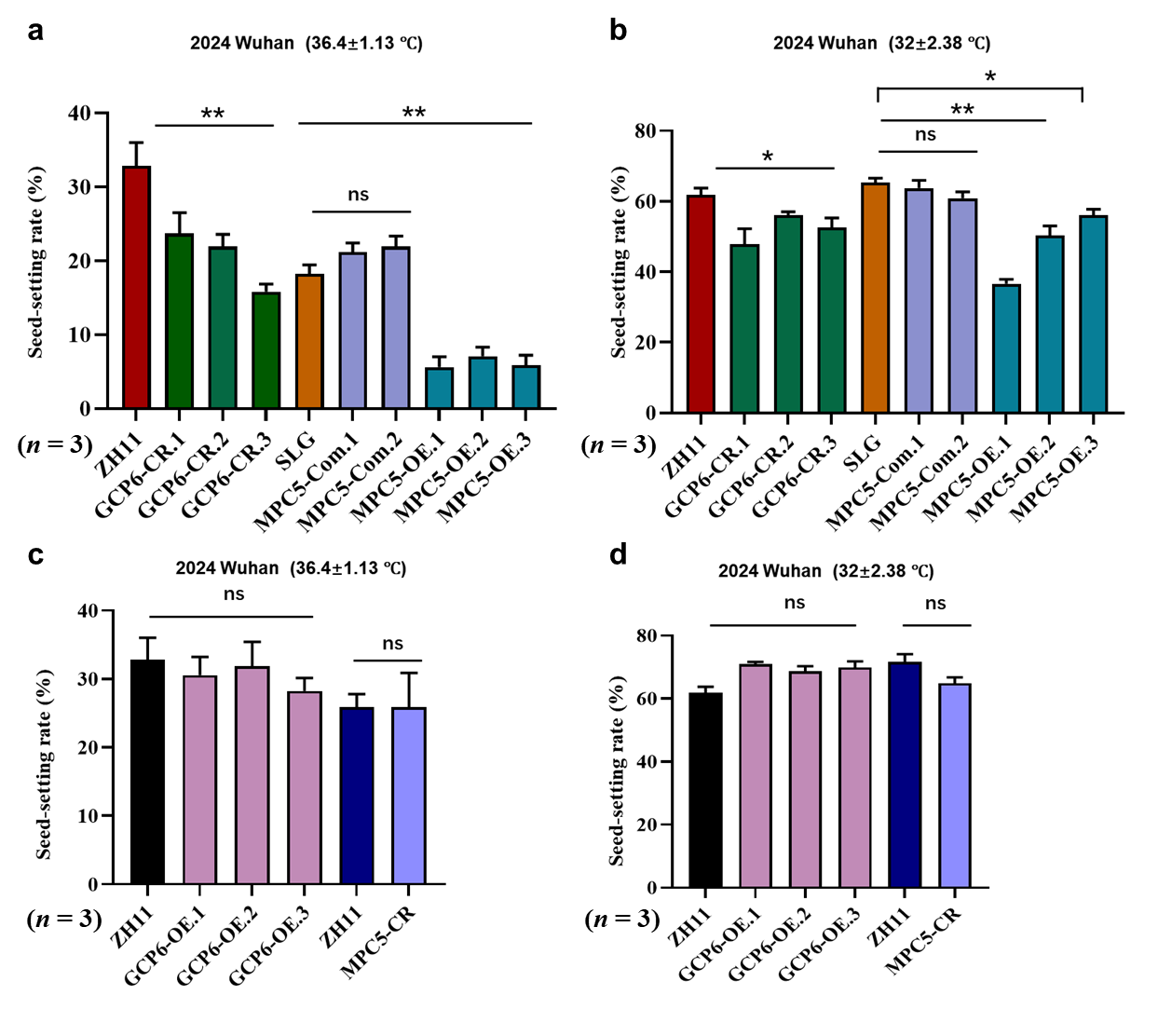


**Figure S13 The seed-setting rate of *MPC5* and *GCP6* transgenic plants. a**,**b**, The seed-setting rate of *GCP6*-CR (**a**) and *MPC5*-OE (**b**) lines at normal and high temperatures. **c**,**d**, The seed-setting rate of *GCP6*-OE (**c**) and *MPC5*-CR (**d**) lines at normal and high temperatures. Three replicates were used to this assay. The statistical significance was generated by two-tailed student’s *t*-test. *, *P* < 0.05; **, *P* < 0.01; ***, *P* < 0.001; ns, not significant.
